# A Multimodal Observational Case-Control Study Exploring Gut Microbiota–Hippocampus Alterations in Individuals With High Positive Schizotypy From the General Population

**DOI:** 10.1016/j.bpsgos.2025.100567

**Published:** 2025-07-17

**Authors:** Galya C. Iseli, Jorge F. Vázquez-Castellanos, David Coynel, James M. Stone, Mariana Zurita Soler, Paul Allen, Fernando Zelaya, Muriel Derrien, Undine E. Lang, Martin Debbané, Ulrich Ettinger, Jeroen Raes, André Schmidt

**Affiliations:** a University of Basel, Department of Clinical Research (DKF), University Psychiatric Clinics, Translational Neurosciences, Basel, Switzerland; b Division of Cognitive Neuroscience, Department of Biomedicine, University of Basel, Basel, Switzerland; c Research Cluster Molecular and Cognitive Neurosciences, Department of Biomedicine, University of Basel, Basel, Switzerland; d Department of Microbiology and Immunology, Rega Institute for Medical Research, Leuven, Belgium; e VIB-KU Leuven Center for Microbiology, Leuven, Belgium; f Brighton and Sussex Medical School, University of Sussex, Brighton, United Kingdom; g Centre for Neuroimaging Sciences, Institute of Psychiatry, Psychology and Neuroscience, King’s College London, London, United Kingdom; h Adult Psychiatric Clinics, University of Basel, Basel, Switzerland; i Faculty of Psychology and Educational Sciences, University of Geneva, Geneva, Switzerland; j Research Department of Clinical, Educational & Health Psychology, University College London, London, United Kingdom; k Department of Psychology, University of Bonn, Bonn, Germany

**Keywords:** Brain-gut axis, Gut microbiota, Hippocampus, Multimodal neuroimaging, Schizotypy

## Abstract

**Background:**

The hippocampus plays a critical role in psychosis, with reduced volume observed across the psychosis continuum. These structural changes are associated with cognitive deficits, symptom severity, and increased risk of psychosis progression. Elevated hippocampal perfusion and glutamate/GABA (gamma-aminobutyric acid) imbalance further suggest metabolic dysregulation as a key mechanism. Gut microbiota composition can influence hippocampal metabolism, but their interplay remains to be explored.

**Methods:**

In this cross-sectional study, we recruited 142 healthy participants from the general population, yielding 69 individuals with high schizotypy (HS) and 72 individuals with low schizotypy. All underwent clinical and cognitive testing, multimodal neuroimaging, and gut microbiota analysis via 16S ribosomal RNA gene sequencing. Hippocampal subfield volumes (structural magnetic resonance imaging), perfusion (arterial spin labeling) and glutamate/GABA levels (proton magnetic resonance spectroscopy), and microbial taxa (abundance, diversity, enterotypes) were assessed.

**Results:**

Group comparisons of cognition, multimodal neuroimaging, and gut microbiome composition did not reveal significant differences after correction for multiple comparisons. Within the HS group, glutamate (*r* = 0.38, *p* = .003) and GABA (*r* = −0.36, *p* = .003) ratios were linked to social withdrawal. Across the entire sample, left hippocampal subfield volumes and glutamate/GABA levels differed significantly between predominant gut microbial enterotypes.

**Conclusions:**

Our results suggest a potential relationship between aberrant gut microbial composition and hippocampal alterations in people with positive schizotypy from the general population. Our findings inform future large-scale research that further explores specific mechanisms of gut microbiome-hippocampus interactions in psychosis and the potential of tailored microbial interventions targeting hippocampal-mediated symptoms.

## Plain Language Summary

In this multimodal study, we investigated the relationship between gut microbiota composition and hippocampal structure, perfusion, and metabolism in individuals with high versus low positive schizotypy from the general population. High schizotypy was associated with subtle cognitive impairments, hippocampal volume differences, altered neurotransmitter ratios, and reduced abundance of short-chain fatty acid–producing gut bacteria. While most group differences did not survive correction for multiple comparisons, the results highlight a possible brain-gut connection in early psychosis risk, suggesting new avenues for future research and prevention strategies.

The hippocampus is a focal point in psychosis research (1). Reduced hippocampal volume is prevalent not only in patients with schizophrenia (SZ) (2, 3, 4) and first-episode psychosis (FEP) (5, 6, 7), but already in individuals at clinical high risk for psychosis (CHR-P) (8, 9, 10, 11, 12) and individuals with subclinical psychotic-like experiences or schizotypal traits (13), suggesting a crucial role during psychosis (14,15). These volume reductions have been linked to deficits in cognitive functioning such as verbal memory performance (16) and global cognition, as well as symptom severity (17, 18, 19), and have proven to be indicative of transition rates into full-blown psychosis (20,21).

Further compelling evidence for hippocampal dysfunction in psychosis originates from the methylazoxymethanol acetate (MAM) model (22,23). Elevated striatal dopamine in the MAM is attributed to excess hippocampal glutamate (Glu) release (22,23). Similarly, human studies have found that elevated hippocampal Glu is associated with overall functioning in unmedicated patients with SZ (2,5,24) and FEP (25) and in CHR-P individuals with subsequent psychosis transition (9,26, 27, 28). According to the MAM model, Glu rises as a result of reduced GABAergic (gamma-aminobutyric acidergic) inhibition of local pyramidal neurons (29), and glutamatergic hyperactivity is proposed to be secondary to GABA dysfunction (30,31). Recently, regional cerebral blood flow (rCBF) has been linked to Glu/GABA imbalance in psychosis (32). Elevated hippocampal CBF has been reported in patients with SZ and FEP, as well as CHR-P individuals and individuals with high schizotypy (HS) (33, 34, 35). Notably, CHR-P individuals with subsequent remission showed normalization of hippocampal CBF (36). Furthermore, a recent study demonstrated that a single oral dose of diazepam can significantly reduce hippocampal hyperperfusion in CHR-P (33). Impairments in hippocampal perfusion (15,34,36,37) and Glu/GABA imbalance (38, 39, 40) have been proposed as key underlying mechanisms that contribute to hippocampal atrophy. While several previous studies have reported reduced hippocampal volume in schizotypy (13,15,41, 42, 43), others have found contradictory results (34,44), although often with elevated CBF (34), which suggests that hyperperfusion may precede volume loss and relate to subclinical symptoms (34) in high-positive schizotypy.

The gut microbiome has been associated with SZ (45), and preclinical work demonstrated modulatory effects on brain functioning such as cognition (46), including memory (47), as well as locomotor activity and anxiety (48). It has been shown that a healthy gut microbiota is necessary for normal hippocampus-dependent cognition and synaptic plasticity (49). The influence of the gut microbiome on hippocampal metabolism is further substantiated by a study that showed altered hippocampal Glu and GABA levels in germ-free mice after receiving a fecal microbiota transplant from patients with SZ (45). A recent study reported that the short-chain fatty acid (SCFA)–producing genus *Lactobacillus* was depleted in drug-naïve patients with SZ compared with healthy control participants, with the effect being reversed after antipsychotic treatment (50). This matches earlier findings of improvement in *Lactobacillus*-treated patients with chronic SZ (51), suggesting the SCFA-producing *Lactobacillus* genus, with its anti-inflammatory properties (52,53), as a potential preventive target.

In this study, we investigated hippocampal volume, perfusion, and Glu/GABA balance and gut microbiota composition in individuals with HS versus low schizotypy (LS) from the general population. Schizotypy reflects subclinical expression of SZ (54,55) and is a widely applied paradigm to investigate etiological factors of SZ spectrum disorders (38,56), which has proven to provide a robust framework to investigate neurobiological mechanisms with the benefit of circumventing confounding effects resulting from comorbidities or antipsychotic medication (34). Based on current findings, we hypothesized lower GABA and higher Glu concentrations, respectively, and increased perfusion in the hippocampus, together with reduced abundance of SCFA-producing bacteria (e.g., *Bifidobacterium*, *Lactobacillus*) in HS compared with LS individuals.

## Methods and Materials

### Participants

In this cross-sectional observational case-control study, we included 142 participants (Table 1) recruited from the general population by screening 2425 healthy individuals for aberrant positive symptoms. Participants were grouped dichotomously and classified as HS or LS (Figure S1) to maximize contrast. Classification into the HS and LS groups was determined using the German translation of the short Oxford-Liverpool Inventory of Feelings and Experiences (sO-LIFE) questionnaire (57,58); individuals who scored ≥6 on the unusual experience (UE) subscale, which is the most validated proxy for positive schizotypy traits in nonclinical samples (34,44,57), were assigned to the HS group, and individuals who scored ≤1 were assigned to the LS group, while continuously ensuring that ≥6 on the UE scale exceeded 1.5 SDs above the screened population median (Figure S1). Groups were matched on age, sex, education, and substance use. Screening details are provided in the Supplement (Table S1). Of the 142 participants who gave informed consent, 1 was excluded afterward, which yielded a final sample of 69 participants with HS and 72 participants with LS (for details, see Figure S2).

**Table 1:** Demographics

	HS, *n* = 69	LS, *n* = 72	Statistic	*p*
Age, Years	22.11 (2.10)	22.48 (1.80)	*U* = 2765	.259
Sex				
Female	50 (72.46%)	50 (69.44%)	χ² = 0.04	.834
Male	19 (27.14%)	22 (30.56%)		
Handedness				
Left	5 (7.25%)	10 (13.89%)	χ² = 2.68	.262
Right	64 (92.75%)	61 (84.72%)		
Ambidextrous	0 (0%)	1 (1.39%)		
Education, Years	13.97 (1.93)	14.39 (1.79)	*U* = 2763	.244
Body Mass Index	22.33 (2.60)	21.65 (2.23)	*U* = 2116	.130
Sports, Hours/Week	3.17 (2.38)	3.14 (2.11)	*U* = 2514	.902
Alcohol				
No	19 (27.54%)	15 (20.83%)	χ² = 0.54	.463
Yes	50 (72.46%)	57 (79.17%)		
Smoking				
No	59 (85.51%)	63 (87.50%)	χ² = 0.01	.921
Yes	10 (14.49%)	9 (12.50%)		
Cannabis				
No	59 (83.33%)	60 (83.33%)	χ² = 0.02	.902
Yes	10 (14.49%)	12 (16.67%)		
IQ	96.96 (8.07)	95.75 (6.41)	*U* = 2222	.343

Values are presented as mean (SD) or *n* (%).

HS, high schizotypy; LS, low schizotypy.

### Clinical Assessment

Psychiatric symptoms were assessed using self-report questionnaires such as the Schizotypal Personality Questionnaire (SPQ) (59,60), the Community Assessment of Psychic Experiences (CAPE) (61,62), the Chapman Scales for Physical and Social Anhedonia (SASPAS) (63,64), the Positive and Negative Affect Schedule (PANAS) (65), the Spielberger State-Trait Anxiety Inventory (STAI) (66), and the Depression Anxiety and Stress Scale (DASS) (67) (Table S2). Furthermore, a regional food frequency questionnaire (68) and the Bristol Stool Scale (BSS) (69) was used as a proxy for transit time (Table S6).

### Cognitive Assessment

Cognition was assessed with the MATRICS Consensus Cognitive Battery (MCCB) (70, 71, 72), which was initially developed as an end point for clinical trials and had previously proven to be highly sensitive in detecting impairment typically observed in SZ (73) and more recently also observed in patients with early psychosis (74) and CHR-P individuals (75) (for details, see the Supplement and Table S2).

### Multimodal Neuroimaging

#### Structural Magnetic Resonance Imaging

All brain measures were collected at the University Hospital Basel using the same MAGNETOM Siemens 3T scanner. Structural magnetic resonance imaging (sMRI) data preprocessing was performed using fMRIPrep 23.0.2, and hippocampus subfield volumes were extracted using the FreeSurfer 7.3+ pipeline recon-all and segment_subregions hippo-amygdala. To control for interindividual variation, all region of interest (ROI) volumes were corrected for intracranial volume using the residual approach (76,77). More detailed imaging protocols are listed in the Supplement.

#### Arterial Spin Labeling

The ASAP toolbox pipeline (78) was used to preprocess all CBF maps. For spatial normalization of the CBF maps to the Montreal Neurological Institute (MNI) space, a multistep approach was used as was done previously in Modinos *et al.* (44). For more details on arterial spin labeling (ASL) processing, see the Supplement. Hippocampal ROIs were selected by applying a mask at the group level of specific areas extracted from the Desikan-Killiany atlas (79).

#### Proton Magnetic Resonance Spectroscopy

Due to reported findings of left hemispheric hippocampal volume reductions in CHR-P (80) and interactions with schizotypal traits (13), proton magnetic resonance spectroscopy (^1^H-MRS) spectra of the left hippocampus were obtained during the same session using water-suppressed Meshcher-Garwood point resolved spectroscopy (MEGA-PRESS) (for details, see the Supplement and Figure S3). Any spectra with a Cramér-Rao lower-bound value >20% were excluded from subsequent analyses (2 Glu, 1 Glu + glutamine [Glx], 17 GABA). Because an unsuppressed water MEGA-PRESS spectrum was not collected, GABA, Glu, and Glx were scaled to *N*-acetylaspartate (NAA) for signal referencing and to correct for partial volume effects prior to analysis. Glu and Glx were also scaled to GABA for the same purpose.

### Microbiota Sequencing, Processing, and Enterotyping

Detailed procedures for stool collection, microbiota sequencing, and quantitative profiling are provided in the Supplement. Alpha diversity was assessed using observed richness, Shannon, and inverse Simpson indices; beta diversity was computed using Bray-Curtis dissimilarities from 16S ribosomal RNA (rRNA) genus-level data (see the Supplement for full analysis details). Bacterial profiles were aggregated at the genus level and integrated with Flemish Gut Flora Project data (*N* = 2998) (81). The gut microbiota was classified into 4 community types (enterotypes) using the Dirichlet-multinomial model to capture ecological patterns beyond individual taxa. This community-level approach, particularly suitable for heterogeneous populations, may reveal novel associations with schizotypy markers despite null findings in single-taxon analyses (81,82). A community-level approach was used to capture broader ecological patterns beyond individual taxa, with enterotypes identified via the Dirichlet-multinomial model. In heterogeneous populations, such frameworks may reveal host-microbiome interactions even when single-taxon analyses show no effects (see the Supplement for details).

### Statistical Analysis

Group differences in clinical, cognitive, and hippocampal measures were tested using independent *t* tests and Mann-Whitney *U* tests for non-normal data and χ^2^ tests for categorical variables. Normality was assessed via Shapiro-Wilk’s test; Student’s or Welch’s *t* tests were applied based on variance equality. Effect sizes were calculated using Cohen’s *d*. Clinical variables that showed significant group differences after correction were correlated with biological measures (hippocampal sMRI, ASL, ^1^H-MRS, microbiome) within the HS group using Spearman correlations. Significant pairs in the HS group were compared with the same pairs in the LS group using Fisher’s *r*-to-*z* transformation, with *p* values corrected per modality via the Benjamini-Hochberg (BH) procedure.

Microbiota profiling used quantitative microbiome profiling including microbial load. To assess the influence of metadata and schizotypy on microbial composition, we conducted distance-based redundancy analysis (db-RDA) at genus and amplicon sequence variant (ASV) levels. Beta diversity between groups was calculated using Bray-Curtis distances and tested via permutational multivariate analysis of variance (PERMANOVA). Enterotype distributions (*Bacteroides* 1/2, *Prevotella*, *Ruminococcus*) were compared using χ^2^ tests. ANCOVA was used to test enterotype-schizotypy effects on clinical outcomes, adjusting for age and stool consistency. Post hoc tests used Mann-Whitney *U* tests with BH correction. All *p* values reflect false discovery rate correction unless otherwise noted. Analyses were performed in R (version 4.1.2).

## Results

### Demographic Characteristics and Nutritional Habits

Demographic and clinical characteristics of the study sample are shown in Table 1. As the sample was prospectively matched, there were no significant group differences in age, sex, or IQ (MWT-B). There were no significant differences between the HS and LS groups in nutritional habits measured by the food frequency questionnaire (83), with effect sizes ranging from Cohen’s *d* = 0.11 to Cohen’s *d* = 0.43 (for uncorrected (unc) and corrected *p* values, see Table S6).

### Clinical Measures and Cognition

**Figure 1:**
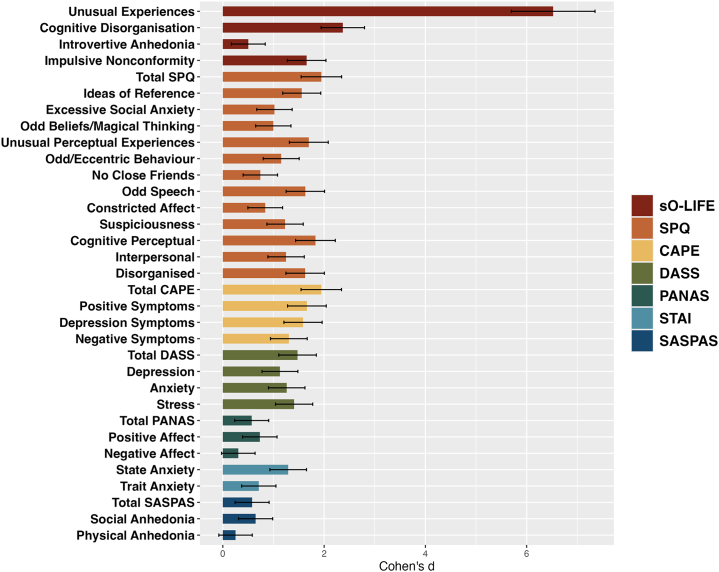
Clinical group differences. Bars represent Cohen’s *d* effect sizes for high schizotypy (HS) vs. low schizotypy (LS) comparisons across clinical measures. Error bars indicate 95% CIs. Positive values reflect greater symptom severity in individuals with HS. CAPE, Community Assessment of Psychic Experiences; DASS, Depression Anxiety and Stress Scale; PANAS, Positive and Negative Affect Schedule; SASPAS, Chapman Scales for Physical and Social Anhedonia; sO-LIFE, short Oxford-Liverpool Inventory of Feelings and Experiences; SPQ, Schizotypal Personality Questionnaire; STAI, State-Trait Anxiety Inventory.

Groups differed on the cognitive disorganization subscale of the sO-LIFE questionnaire (*p* < .001, Cohen’s *d* = 2.37) and the impulsive nonconformity subscale (*p* < .001, Cohen’s *d* = 1.65), whereas the introvertive anhedonia subscale (*p* = .113, Cohen’s *d* = 0.50) showed a moderate effect size, but the groups did not differ after BH correction. Total scores on the SPQ (*p* < .001, Cohen’s *d* = 1.95), CAPE (*p* < .001, Cohen’s *d* = 1.94), DASS (*p* < .001, Cohen’s *d* = 1.48), SASPAS (*p* < .028, Cohen’s *d* = 0.58), and PANAS (*p* < .001, Cohen’s *d* = 0.57), as well as both STAI state (*p* < .001, Cohen’s *d* = 1.29) and trait (*p* < .001, Cohen’s *d* = 0.71) anxiety scores, differed significantly between groups after BH correction, with large effect sizes. A comprehensive list of clinical group differences including all subscores and corrected *p* values is provided in Figure 1 and Table S3.

**Figure 2:**
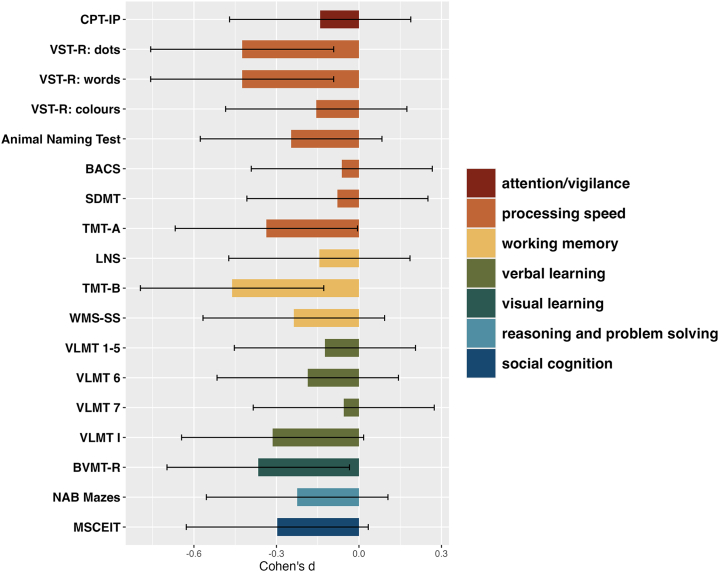
Cognitive group differences. Bars show Cohen’s *d* effect sizes for the high schizotypy (HS) vs. low schizotypy (LS) comparisons across cognitive tests. Error bars represent 95% CIs. Negative values reflect poorer performance in individuals with HS. BACS, Brief Assessment of Cognition in Schizophrenia; BVMT-R, Brief Visuospatial Memory Test-Revised; CPT-IP, Continuous Performance Test-Identical Pairs; LNS, Letter-Number Span Test; MSCEIT, Mayer-Salovey-Caruso Emotional Intelligence Test; NAB, Neuropsychological Assessment Battery; SDMT, Symbol Digit Modalities Test; TMT, Trail Making Test; VLMT, Verbal Learning Memory Test; VST-R, Victoria Stroop Test after Regard; WMS-SS, Wechsler Memory Scale-Spatial Span.

Cognitive data showed trends for group differences in selective attention, processing speed, working memory, visuospatial memory, verbal learning, and social cognition, with participants in the HS group performing more poorly than participants in the LS group (see Figure 2). However, these differences did not survive correction for multiple comparisons (see the Supplement and Table S4).

### Hippocampal Volume, Perfusion, and Metabolism Alterations in the HS Group

**Figure 3:**
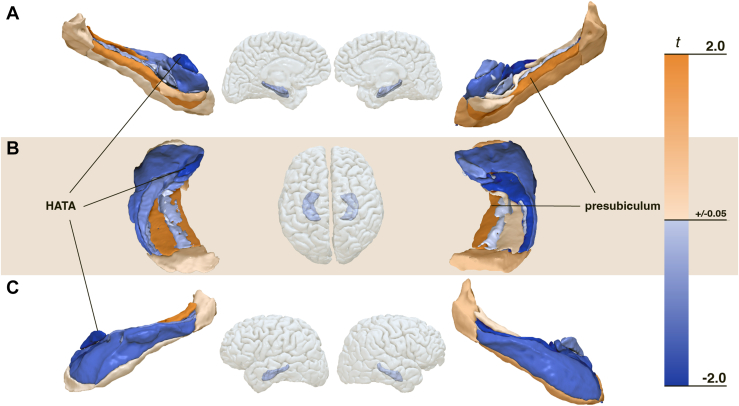
Visualization of hippocampal subfield volume differences between high schizotypy (HS) and low schizotypy (LS) groups. The left and right panels show hippocampal subfields from different hemispheres: **(A)** medial view, **(B)** superior view, and **(C)** anterior view. Subfield segmentation was performed using FreeSurfer version 6.0. The center panel displays hippocampal volume differences between groups. The color gradient reflects *t* values from group comparisons, where blue indicates lower volumes in HS than LS (*t* < −0.05), and orange indicates higher volumes in HS than LS (*t* > 0.05) groups.

**Figure 4:**
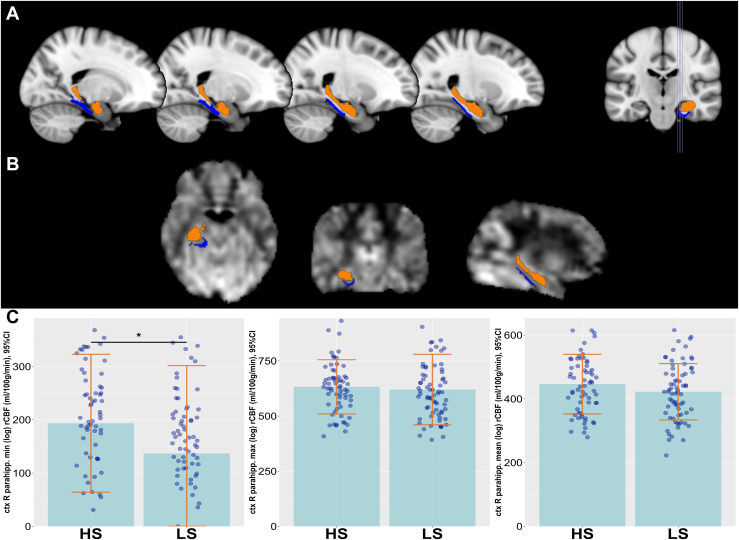
Regional cerebral blood flow (rCBF) differences in the right hippocampus. **(A)** Region of interest mask for the right hippocampus (orange) and right parahippocampal cortex (ctx R parahipp.) (blue), overlaid on a standard brain T1 template and **(B)** on a representative subject-level CBF map in normalized space, and **(C)** groupwise comparison of rCBF (log-transformed, in mL/100 g/min) in the right parahippocampal cortex (minimum, maximum, and mean values) in individuals with high schizotypy (HS) and low schizotypy (LS). Bars represent group means; error bars indicate ±1 SD. Individual data points are shown as jittered dots to illustrate within-group variation. ∗significance level <.05 before Benjamini-Hochberg correction.

Hippocampal group differences did not withstand corrections for multiple comparisons. Volumetric subfield analysis showed statistical trends for increased right presubiculum body (*p*_unc_ = .081, Cohen’s *d* = 0.30) and reduced left hippocampus amygdala transitional area (HATA) (*p*_unc_ = .066, Cohen’s *d* = 0.32) in the HS group (Figure 3; see Table S5 for corrected *p* values). No group differences were found in global perfusion or hippocampal GABA or Glu concentrations (see Table S5). Without BH correction, individuals with HS showed higher minimum perfusion in the right parahippocampal cortex (*p*_unc_ = .027, Cohen’s *d* = 0.38) (Figure 4). Full statistics including corrected *p* values are reported in Table S5.

Subsequent post hoc power analyses based on observed effect sizes were conducted to estimate the sample sizes required to detect robust group differences using multiple comparison correction.

The analyses indicated that a minimum of 157 participants per group would be necessary to detect differences in hippocampal volume, and more than 109 per group would be needed for CBF, at corrected significance thresholds.

### Associations Between Symptoms and Hippocampus Markers in HS

**Figure 5:**
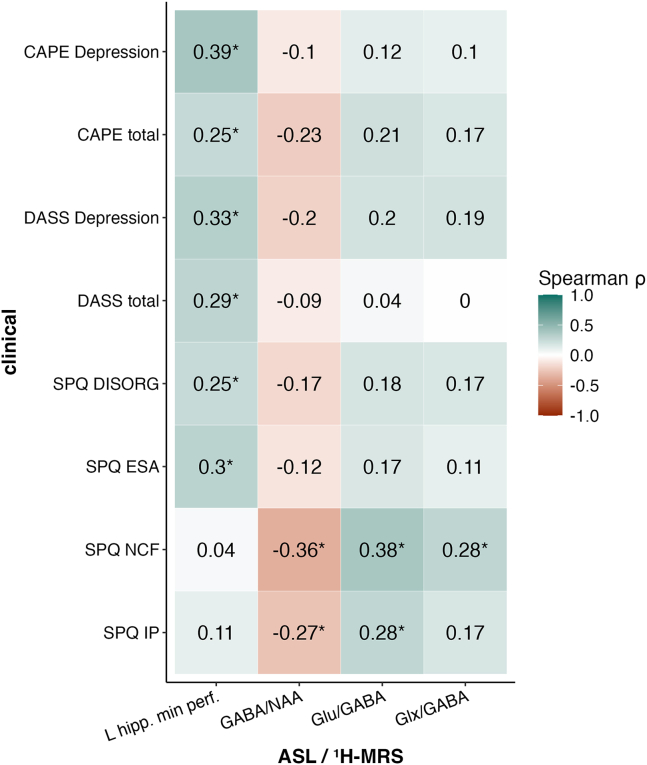
Heatmap showing Spearman with Fisher’s *z* transformation correlation coefficients within high schizotypy (HS) that differ significantly from low schizotypy (LS). Clinical variables on the y-axis and left hippocampal (L hipp.) biological markers: arterial spin labeling (ASL) and proton magnetic spectroscopy (^1^H-MRS). ∗Indicate significant differences between groups after corrections. CAPE, Community Assessment of Psychic Experiences; DASS, Depression Anxiety and Stress Scale; DISORG, disorganized; ESA, excessive social anxiety; GABA, gamma-aminobutyric acid; Glu, glutamate; Glx, glutamate + glutamine; IP, interpersonal; NAA, *N*-acetylaspartate; NCF, no close friends; perf., perfusion; SPQ, Schizotypal Personality Questionnaire.

The correlation analysis between clinical symptom scores and hippocampal measures revealed significant differences in perfusion levels and neurotransmitter concentrations in the left hippocampus between groups after corrections. Within the HS group, left hippocampal minimum perfusion levels showed positive correlations with the CAPE total (*r*_65_ = 0.25, *p*_diff_ = .018) and depression (*r*_65_ = 0.39, *p*_diff_ = .016) scores, the DASS total (*r*_66_ = 0.29, *p*_diff_ = .002) and depression (*r*_66_ = 0.33, *p*_diff_ = .003) scores, and the SPQ disorganized (*r*_65_ = 0.25, *p*_diff_ = .006) and excessive social anxiety (*r*_65_ = 0.30, *p*_diff_ = .002) subscales. A negative correlation between the SPQ interpersonal (IP) scale (*r*_59_ = −0.27, *p*_diff_ = .041) and the no close friends (NCF) scale (*r*_59_ = −0.36, *p*_diff_ = .003) was found with left hippocampal GABA/NAA concentrations. In addition, positive correlations were found between Glu/GABA and Glx/GABA concentrations and the same SPQ subscales: IP (*r*_59_ = −0.27, *p*_diff_ = .041) and NCF (*r*_59_ = −0.27, *p*_diff_ = .041) (Figure 5 and Figure S4). No significant differences were detected in hippocampal volume or microbiome abundance.

### Associations Among Hippocampal Markers in the Total Sample

Negative correlations between the left hippocampal tail volume and the maximal perfusion level (*r*_131_ = −0.20, *p*_unc_ = .022) and positive correlations between minimum hippocampal perfusion levels and Glu/NAA ratios (*r*_132_ = 0.019, *p*_unc_ = .027) and between left hippocampal parasubiculum volumes and GABA/NAA ratios (*r*_119_ = −0.19, *p*_unc_ = .038) (Figure S5) were found, but they did not survive correction for multiple comparisons (Table S5).

### Gut Microbiota Ecosystem-Level Analysis

No significant differences in gut microbiota composition were observed between the HS and LS groups at the genus or ASV level. The BSS, which served as a proxy for transit time, contributed significantly to compositional variation. Enterotype distribution and alpha diversity metrics (species abundance, distribution, richness, and total microbial load) also did not differ between groups (Figures S6 and S7; Table S6). Full statistical results and figures are provided in the Supplement.

### Associations Between Gut Microbiota Taxa, Schizotypy, and Hippocampal Volumes

Differential analysis comparing individuals with HS and LS was conducted in the total sample. The quantitative abundance of 4 genera differed between the HS and LS groups, although the differences did not survive multiple testing correction: *Gordonibacter* (Mann-Whitney; *z* = −2.23, *p*_unc_ = .006), *Eubacterium D* (Mann-Whitney; *z* = −2.09, *p*_unc_ = .031), *Alistipes A* (Mann-Whitney; *z* = −1.89, *p*_unc_ = .046), and *Anaerorhabdus* (Mann-Whitney; *z* = −1.62, *p*_unc_ = 0.046), all of which were lower in the HS group than the LS group (see Figure S8; for corrected *p* values, see Table S7). Of these, genus *Gordonibacter* showed a negative correlation with the SPQ paranoid ideation subscale (*r* = −0.18, *p*_unc_ = .038), and genus *Anaerorhabdus* showed a positive correlation with left hippocampal presubiculum head volumes (*r* = 0.18, *p*_unc_ = .038) before correction.

### Association Between Gut Enterotypes and Hippocampal Markers Across the Entire Sample

**Figure 6:**
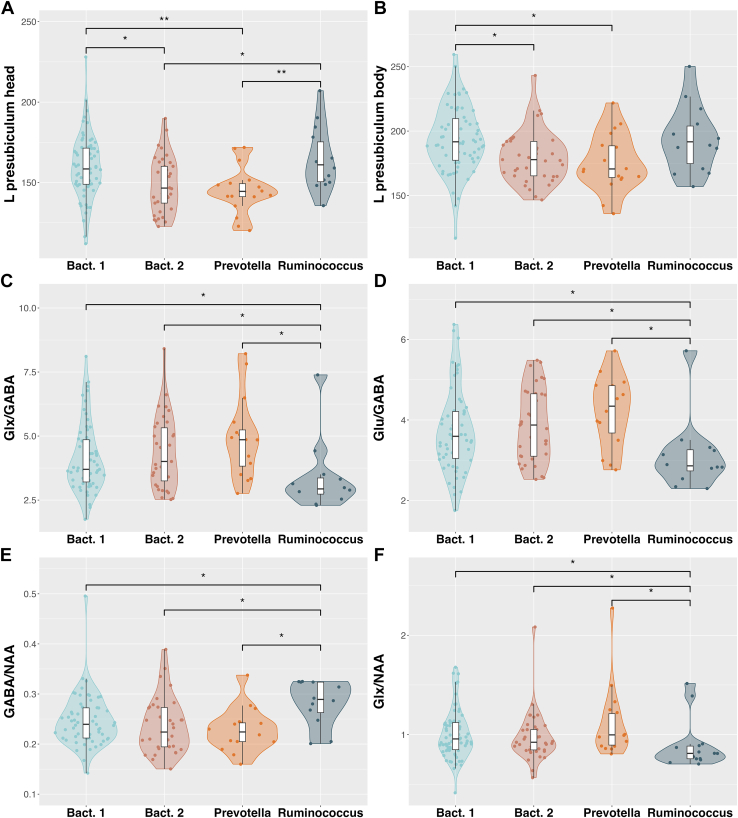
Enterotype differences of left hippocampal volumes and proton magnetic spectroscopy neurometabolite ratios. Violin plots showing the distribution of hippocampal markers across enterotype groups (*Bacteroides* 1 [Bact. 1], *Bacteroides* 2 [Bact. 2], *Prevotella*, *Ruminococcus*). Boxplots represent group medians and IQRs, while individual data points are displayed as sina plots to illustrate participant-level variation. Asterisks indicate statistically significant group differences (∗*p* < .05, ∗∗*p* < .01) based on pairwise comparisons using Mann-Whitney *U* tests (Benjamini-Hochberg corrected). GABA, gamma-aminobutyric acid; Glu, glutamate; Glx, glutamate + glutamine; L, left; NAA, *N*-acetylaspartate.

Due to significant differences between enterotypes in age (*F*_3,137_ = 3.01, *p* = .03) and BSS consistency (*F*_3,136_ = 2.69, *p* = .05), they were included as covariates in subsequent analyses. Investigating the relationship between enterotypes and hippocampal volumes in the total sample revealed statistically significant differences between *Bacteroides* 1 and *Bacteroides* 2 (*U* = 1647, *p* = .011, *r* = 0.66), *Bacteroides* 1 and *Prevotella* (*U* = 903, *p* = .007, *r* = 0.75), and *Prevotella* and *Ruminococcus* (*U* = 45, *p* = .007, *r* = 0.18) for left presubiculum head volumes (*F*_3,__127_ = 5.89, *p* = .019), and between *Bacteroides* 1 and *Bacteroides* 2 (*U* = 1700, *p* = .011, *r* = 0.69), and between *Bacteroides* 1 and *Prevotella* (*U* = 857, *p* = .019, *r* = 0.71) for left presubiculum body volumes (*F*_3,127_ = 4.71, *p* = .041) (Figure 6).

^1^H-MRS data indicate that the neurotransmitter concentrations of Glx/GABA (*F*_3,111_ = 4.69, *p* = .011), GABA/NAA (*F*_3,111_ = 3.74, *p* = .022), Glu/GABA (*F*_3,111_ = 4.59, *p* = .011), and Glx/NAA (*F*_3,111_ = 2.88, *p* = .048) in the left hippocampus correlate with enterotype. Pairwise comparisons specifically suggest that the *Ruminococcus* enterotype is related to Glu and GABA neurotransmitter concentrations in the left hippocampus (Figure 6 and Table S8). The interaction effect between schizotypy and enterotype approached statistical significance only in predicting SPQ paranoid ideation scores, with a marginal trend observed (*F*_3,128_ = 1.06, *p* = .070).

## Discussion

This study is the first to investigate hippocampus volume, perfusion, and metabolism and link them to gut microbial composition and cognitive as well as clinical data in a large schizotypy sample. When focusing on results that survive correction for multiple comparisons, 3 major findings emerge. Firstly, HS individuals exhibited significantly elevated symptom severity across a broad range of clinical domains. These included cognitive disorganization, impulsive nonconformity, depression, anxiety, and stress; they also had higher scores on measures of all schizotypal traits, subclinical psychotic experiences, and both state and trait anxiety. Secondly, within the HS group, we observed significant associations between neurotransmitter imbalance in the left hippocampus and social withdrawal symptoms. Specifically, elevated Glu/GABA and Glx/GABA ratios and reduced GABA/NAA ratios were correlated with the SPQ NCF and IP subscales. Thirdly, gut microbiome composition at the community level (enterotypes) was significantly associated with hippocampal structural and metabolic markers. Specifically, enterotypes differed in their relationship to left hippocampal presubiculum head and body volumes, as well as Glx/GABA, Glu/GABA, GABA/NAA, and Glx/NAA ratios.

The robust clinical group differences, observed across multiple clinical instruments, substantiate the validity of the HS construct and its association with broad subclinical psychiatric symptomatology. Notably the two groups differed significantly across all traits measured by the SPQ including the subscales NCF, excessive social anxiety, and constricted affect, which are traits attributed to negative symptoms. However, the groups did not differ significantly on anhedonia subscales, which suggests that social withdrawal may precede the emergence of anhedonic symptoms. This highlights social withdrawal as a potential early target for interventions addressing excitatory-inhibitory (E/I) imbalance (84). Consistent with previous studies in HS (38) and unmedicated FEP (85), we did not find significant group differences in Glu or GABA levels. However, the Glu/GABA ratio positively correlated with the SPQ subscale NCF in the HS group, differing significantly from the LS group after correction. These findings are consistent with earlier research linking Glu/GABA imbalance to impaired social functioning in the general population (86), highlighting the potential neurochemical basis of social deficits in schizotypy. Given the limited efficacy of pharmacological treatments for addressing negative symptoms and cognitive impairments (87), key predictors of quality of life and social functioning in individuals with SZ (84), the microbiome, which has recently been shown to influence both cognitive functions (88) such as working memory and social behavior (89), emerges as another promising treatment target.

Microbiome involvement is further substantiated by our findings showing differences in enterotype associations with left presubiculum volumes and neurochemical ratios that reflect E/I imbalance and neurometabolite profiles. Notably, individuals classified as *Ruminococcus* enterotype exhibited patterns suggestive of neuroprotective profiles, including higher presubiculum volumes and more favorable neurotransmitter ratios compared with individuals classified as *Prevotella* or *Bacteroides* 2 enterotypes. These differences in GABA/NAA, Glu/GABA, Glx/GABA, and Glx/NAA between enterotypes suggest the microbiome composition as a potential target for the disrupted neurometabolite-brain activity observed at later stages on the psychosis continuum (90). The previously linked pathophysiology of negative symptoms, with reduced Ruminococcaceae and an increase in *Bacteroides* (91), further implicates the gut microbiome. Notably, Ruminococcaceae reductions have also been observed in bipolar disorder (92) and major depressive disorder (93), suggesting a protective role for this microbial family in maintaining mental health. These results highlight the potential of the gut microbiome, particularly *Ruminococcus*, in modulating brain structure and neurochemistry. As psychotropic drugs may increase gut dysbiosis, future gut-targeted interventions using gut-neutral antipsychotics and microbiome profiling could reduce side effects and improve treatment efficacy (94).

While our core conclusions are based on findings that survived multiple comparison correction, several notable trends emerged that, although statistically nonsignificant, may nonetheless inform hypotheses for future research.

Consistent with CHR-P studies (95), no significant hippocampal volume differences between the HS and LS groups survived correction. However, the trend toward larger right presubiculum volume in HS is consistent with studies that have linked increased hippocampal volume to psychosis transition (96). Similarly, reduced left HATA volumes in HS are consistent with recent reports of inward morphological deformation in the hippocampal head and tail, as well as the amygdala, which have been associated with greater severity of positive symptoms in SZ (97). Based on our results, future studies would require sample sizes of >157 participants per group to reliably detect HATA differences under corrected thresholds.

A recently published longitudinal study identified that diminished hippocampal tail volume was prevalent in both CHR-P and FEP (12). In our study, a negative correlation between hippocampal tail volumes and maximal perfusion levels was observed in the total sample, although it did not survive multiple testing correction.

As in previous CHR-P studies (98), we found no significant mean rCBF difference. However, the trend toward higher right parahippocampal minimum perfusion in HS is consistent with reports of elevated right hippocampal rCBF in CHR-P with poor outcomes (28) and in ultra-high-risk individuals (36,37). Hippocampal hyperactivity has become a marker of adverse outcome (28,99) and has emerged as a novel pharmacological treatment target (100). To investigate whether hippocampal perfusion levels are already altered in individuals with high positive schizotypy, future studies would, based on our results, require a sample size of >109 per group to be sufficiently powered.

Genera that produce SCFAs such as butyrate may play a key role in regulating inflammation (101). Specifically, *Eubacterium D*, which showed depletion in our HS group before corrections, has been reported as being depleted in SZ and positively correlated with gray matter volume alterations of the hippocampus, insula, and thalamus (101), suggesting structural brain changes in SZ being driven by inflammation (102). Rifaximin (103), a gut-specific antibiotic, was shown to increase *Eubacterium* and induce significant improvements in cognition, specifically in working memory (104). *Alistipes*, depleted in SZ (101) and LS before corrections, indicate that certain microbial genera may have a protective function provided by their anti-inflammatory properties, thus potentially mitigating inflammatory-related damage.

There is wide consensus on the importance of elucidating the pathogenesis of psychosis using multivariate and data-driven approaches to detect latent biotypes linked to disease progression and treatment response (84). Our results support the hypothesis that the gut microbiome may play a role during early stages of psychosis and holds potential as both a prophylactic and a therapeutic target (105). However, its effects on hippocampal structure, function, and symptom-specific mechanisms require further large-scale investigations (104).

Our findings must be interpreted in light of several limitations. First, the exploratory nature and limited sample size of the study reduce the robustness of the results. Most effects did not withstand multiple comparison correction, which underscores the need for caution in interpretation. These preliminary findings should be considered hypothesis generating and warrant replication in larger, sufficiently powered studies. Second, the ≥6 threshold for HS limits comparability with studies that have used other cutoffs (14,38), underscoring the need for replication using standardized criteria.

Furthermore, due to the absence of blood sample collection and the use of 16S rRNA sequencing, which does not directly quantify SCFA concentrations, all indications of SCFA abundance in this study are indirect and limited to inferred production potential based on microbial composition. A combined approach using shotgun metagenomics and metabolomics would provide a more comprehensive and functional insight into SCFA dynamics; however, this was beyond the scope of the current study. Lastly, the cross-sectional design precludes conclusions about schizotypy trajectories or progression to clinical psychosis. Longitudinal studies with follow-up are needed to assess links with transition rates. Despite these limitations, the study has notable strengths. To our knowledge, this is the largest single-site schizotypy sample with multimodal neuroimaging and microbiome data. Participants were matched on key demographic variables and substance use, and individuals with recent medication intake (including anti-inflammatories); comorbidities; or family histories of schizoaffective, bipolar, or autism spectrum disorders were excluded. These exclusions reduced confounding from medication or psychiatric comorbidity but may limit generalizability to clinical cohorts.

### Conclusions

This study marks the first attempt to elucidate gut microbiota–hippocampus alterations in a healthy sample with subclinical psychotic symptoms from the general population. Our results reveal a modest link between the gut microbiome and hippocampal markers associated with psychosis in a preclinical sample, which warrants larger studies. Future research using dimensional data and advanced multivariate methods, including machine learning, could expand correlation-based analyses by offering a more integrated multidimensional view. These approaches may clarify complex interactions between the gut microbiome, hippocampal structure and function, and psychosis traits, paving the way for novel prevention and treatment strategies.

## Supplementary Material

Supplemental Methods, Results, Figures S1–S9, and Tables S1–S8

Key Resources Table
