# Supplementary material for: A Multimodal Observational Case-Control Study Exploring Gut Microbiota–Hippocampus Alterations in Individuals With High Positive Schizotypy From the General Population
Source: Biol Psychiatry Glob Open Sci. 2025 Jul 17;5(6):100567. doi: 10.1016/j.bpsgos.2025.100567 (PMC12408256; doi:10.1016/j.bpsgos.2025.100567)
Supplement: Supplemental Methods, Results, Figures S1–S9, and Tables S1–S8 [file mmc1.pdf]

SUPPLEMENTARY INFORMATION

A Multimodal Observational Case-Control Study Exploring Gut Microbiota – Hippocampus Alterations in Individuals with High Positive Schizotypy from the General Population

Iseli *et al.*

1. Methods

1.1 Recruitment and sample characteristics

The study was advertised via advertisement boards, in public transportation, in newsletters, sent via email to university students, posted online on websites and social media platforms. Individuals interested in study participation contacted the recruitment team via e-mail upon which an online screening questionnaire was sent out. Due to lack of consensus on a positive schizotypy threshold, we continuously re-evaluated the determined cut-off for the HS group by averaging the UE score only including individuals scoring at least 1.5 SD above the calculated median. After an initial online screening where group allocation was determined, potentially eligible participants were contacted for a phone screening during which the MINI international neuropsychiatric interview was performed excluding participants with the indication of another psychopathology. The following exclusion criteria were applied: exercise >8h per week, pregnancy, lactation, medication intake less than one month before the trial, central nervous system medication intake, tobacco dependence, daily cannabis consumption, probiotics intake, antibiotics intake three months prior to study visit, diet change three months prior to study, headache disorder, gastrointestinal disorder, appendicitis resulting in appendectomy, and first-degree family history of schizophrenia, schizoaffective disorders, bipolar disorder, autism spectrum disorder or suicide. Additionally, participants were screened for MRI eligibility.

Table S1: Exclusion Criteria

Category	Exclusion criteria
demographics	Age < 18 or > 25<br>Not German speaking<br>Body mass index < 18 or > 30<br>Exercise > 8h/week<br>Pregnant or lactating in women
sO-LIFE classifier criteria	Intermediate scores on UE >1 or < 6<br>LS scoring too high on other subscales<br>(CD >11, IA > 7, IN > 7)
medication intake	regular intake of medication (less than one month from the last intake)<br>central nervous system medications (antidepressants, sedatives, or anxiolytics)<br>opioid analgesics
substance intake	tobacco dependence (daily smoking)<br>daily cannabis consumption
microbiota-modifying substances intake	Probiotics or magnesium in the month prior to the second study visit<br>Antibiotics three months prior to the second study visit<br>Diet three months prior to the study
psychiatric disorders	Any personal acute psychiatric disorder<br>First-degree family history of schizophrenia, schizoaffective disorders, bipolar disorder, autism spectrum disorder or suicide
somatic contraindication	Headache disorder<br>Gastrointestinal disorder (eg. irritable bowel syndrome)<br>Active pain disorder<br>Appendicitis resulting in appendectomy
MRI contraindication	Metallic implants or splinters<br>Pacemaker<br>Claustrophobia<br>Tattoos (large spaces of black ink or inked recently)

Figure S1: Recruitment Population

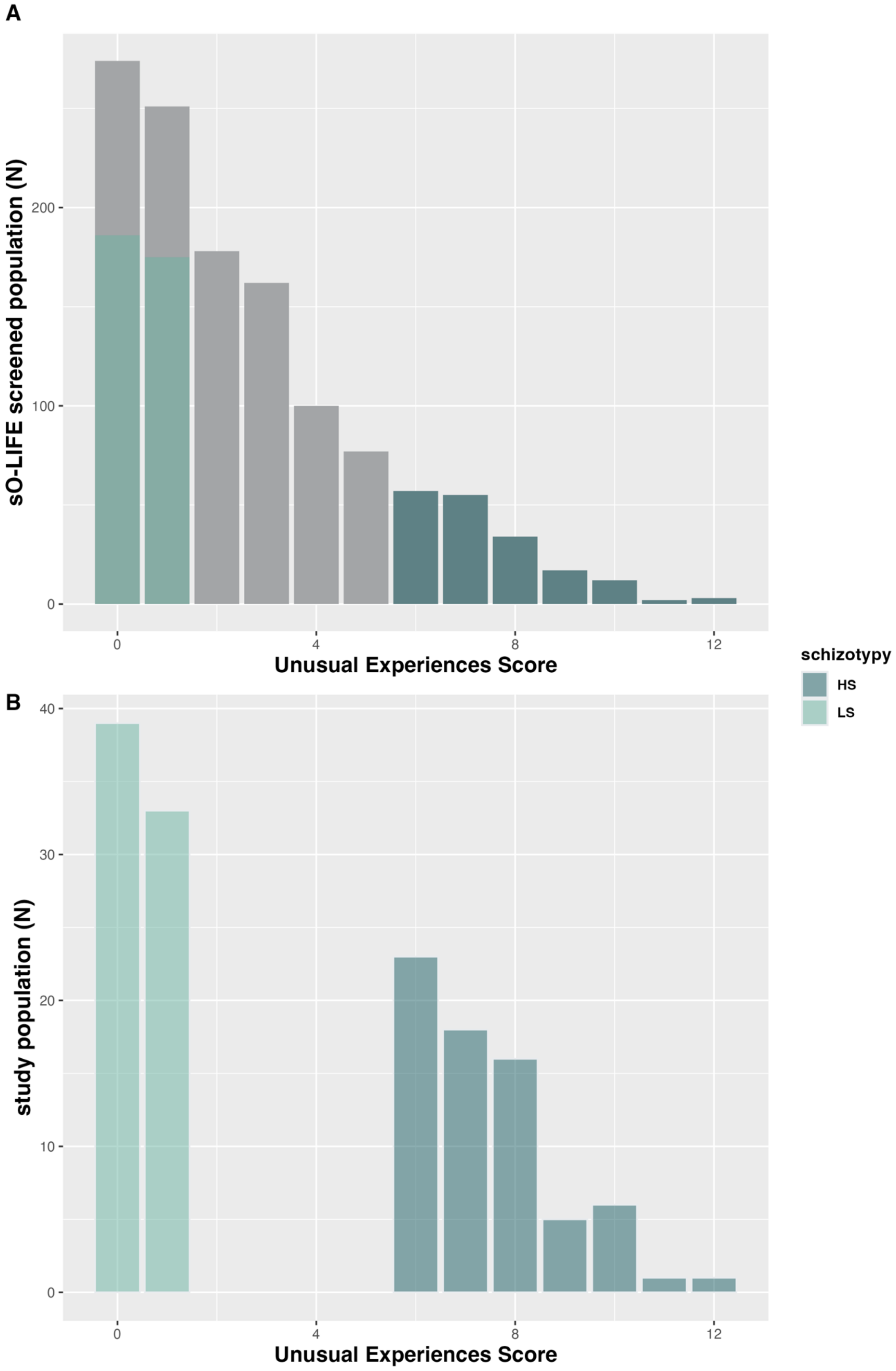

Figure S2: Recruitment Flowchart

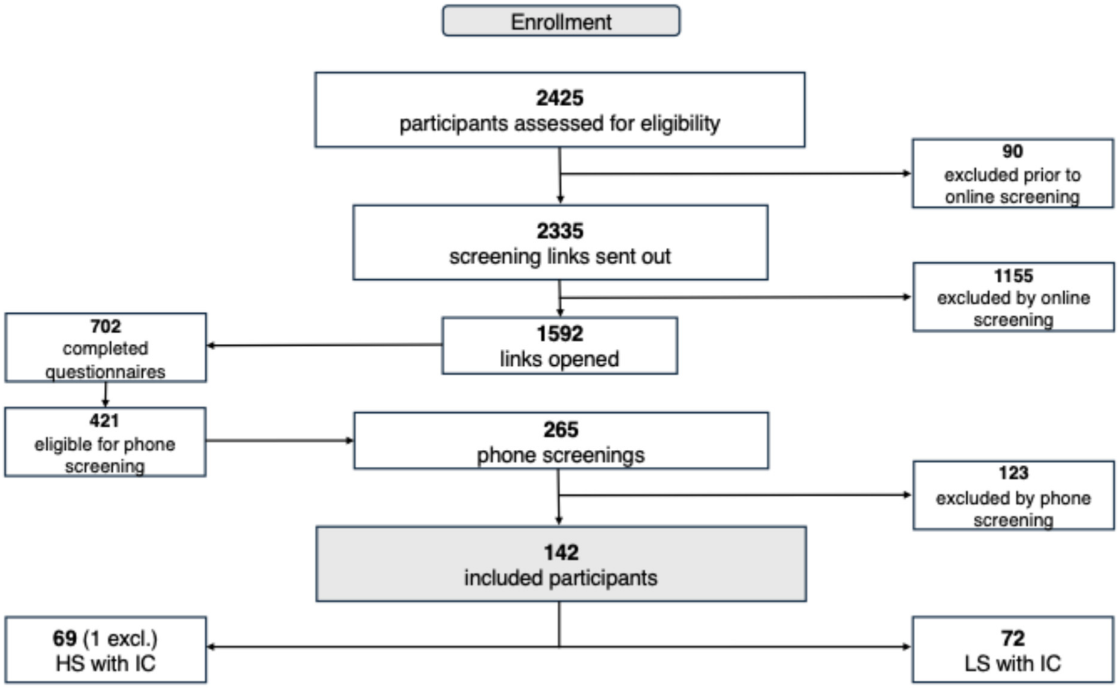

1.2 Study procedure visits 1 & 2

If the participants were eligible, they were invited for two study visits. During the first visit at the University Psychiatric Clinics Basel, after a brief MRI eligibility screening and informed consent, participants were asked to fill out several clinical self-report questionnaires about their perception of experienced anxiety, stress, depression, negative affect, social anhedonia and psychic experiences. Following the digitally administered questionnaires, IQ was evaluated utilising paper-pencil-based tests. After testing, participants were accompanied to the EEG lab, where task-based EEG scans were performed. After the scan, participants were handed the stool sample collection kit and instructed on the extraction method via instruction video. This video was sent to them after the first visit, and they were instructed to return the stool samples during the second visit at the University Hospital Basel. The second study visit took place between one day and one month after the first study visit, where some nutritional and clinical self-report questionnaires were completed, followed by an extensive cognitive test battery. The MRI scan was conducted afterwards, following another screening check for MRI eligibility. The scan consisted of a structural T1 weighted mprage, a resting state fMRI scan, an arterial spin labelling protocol followed by MRS scans for glutamate and GABA with a voxel placement

of the hippocampus in the left hemisphere. The scanning protocol continued for an hour after which participants were compensated for their study participation.

1.3 Cognitive assessments

The MATRICS assesses the domains of processing speed (ANT, BACS, TMT-A), attention/vigilance (CPT-IP), working memory (WMS-III, LNS), verbal learning (VLMT), visuospatial memory (BVMT-R), reasoning and problem-solving (NAB), and social cognition (MSCEIT). The MATRICS original verbal learning task (HVLT-R) was replaced with the Verbal Learning Memory Task (VLMT(1, 2)) more commonly used in German-speaking cohorts. Additionally, we included tests for verbal IQ (MWT-B(3)), selective attention and response inhibition (Stroop(4)/VST-R(5)), executive function (TMT-B(6)), and processing speed (SDMT(7)).

Table S2: Clinical and cognitive assessments

assessment type	assessment domain	assessment name	assessment description
Questionnaire	clinical symptoms	Community Assessment of Psychic Experiences **(CAPE)(8, 9)**	42-item self-report questionnaire for psychotic-like experiences.(10, 11)
		Depression, Anxiety and Stress Scale **(DASS)(12)**	42-item self-report questionnaire designed to measure negative emotional states of depression, anxiety and stress.
		The Positive Affect Negative Affect Schedule **(PANAS)(13)**	20-item self-report questionnaire consisting of 10-item scales to measure positive and negative affect.(14)
		The Chapman Scales for Physical and Social Anhedonia **(SASPAS)(15, 16)**	43-item self-report questionnaire assessing physical and social anhedonia.
		The Oxford-Liverpool Inventory of Feelings and Experiences, short scales **(sO-LIFE)**(17, 18)	30-Item self-report questionnaire describing unusual experiences (UE) in 12 items, cognitive disorganisation (CD) in 11 items, introvertive anhedonia (IA) in 10 items, and impulsive nonconformity (IN) in 10 items.(19)
		Schizotypal Personality Questionnaire **(SPQ)(20, 21)**	74-item self-report questionnaire assessing nine subscales of schizotypal personality.<br>Subscales:<br>ideas of reference<br>excessive social anxiety<br>odd beliefs/magical thinking<br>unusual perceptual experiences<br>odd or eccentric behaviour<br>no close friends<br>odd speech<br>constricted affect<br>suspiciousness/paranoid ideation<br>cognitive perceptual<br>interpersonal<br>disorganised
		The Spielberger Trait Anxiety Inventory **(STAI)(22)**	40-item self-report questionnaire assessing state and trait anxiety.
	gastrointestinal symptoms	Nutritional and Digestion Questionnaire	Questions on nutrition and bowel movements.
		Food Frequency Questionnaire **(FFQ)(23)**	Regionally validated food frequency questionnaire.(24)
		Bristol Stool Scale **(BSS)(25)**	Diagnostic medical tool designed to classify the form of human faeces into seven categories, measuring consistency and intervals.(26)

**Test**	**cognition**	Fluency: Animal Naming Test (**ANT**)(27)	Oral test in which respondent names as many animals as they can in 1 minute.(28)
		Brief Visuospatial Memory Test Revised **(BVMT-R)(29)**	A test that involves reproducing six geometric figures from memory.(28)
		Brief Assessment of Cognition in Schizophrenia **(BACS)**: Symbol-Coding(30)	Timed paper-pencil test which respondent uses a key to write digits that correspond to nonsense symbols.(28)
		Continuous Performance Test – Identical Pairs (**CPT-IP**)(31)	Computer-administered measure of sustained attention in which respondent presses a response button to consecutive matching numbers.(28)
		Letter-Number Span (**LNS**)(32)	Orally administered test in which respondent mentally reorders strings of number and letters and repeats them to administrator.(28)
		Mayer-Salovey-Caruso Emotional Intelligence Test (**MSCEIT**^{TM}): Managing Emotions(33)	Multiple-Choice test that assesses how people manage their emotions.
		Mehrfach-Wortschatz-Intelligenztest **(MWT-B)(3)**	Evaluation of verbal intelligence.
		Neuropsychological Assessment Battery (NAB®): **Mazes(34)**	Seven timed paper-pencil mazes of increasing difficulty that measure foresight and planning.(28)
		Symbol-Digit-Modalities-Test (**SDMT**)(7)	Screening for cerebral dysfunction in children and adults.
		Victoria Stroop Test adapted by Regard **(VST-R)** (4, 35)	Assessment of ability to inhibit cognitive inference.
		Trail Making Task A (**TMT-A**)(36, 37)	Timed paper-pencil test in which respondent draws a line to connect consecutively numbered circles placed irregularly on a sheet of paper.(28)
		Trail Making Task B (**TMT-B**)(36, 37)	Timed paper-pencil test in which respondent draws alternating a line to connect consecutive numbers and alphabetical letters in circles placed irregularly on a sheet of paper
		Verbaler Lern- und Merkfähigkeitstest (**VLMT**)(2)	Verbal learning and memory test.
		Wechsler Memory Scale® - 3rd Ed.: Spatial Span (**WMS®-III)**(38)	Using a board on which 10 cubes are irregularly spaced, respondent taps cubes in same (or reverse) sequence as test administrator.(28)

1.4 Structural MRI: data acquisition and preprocessing

MRI scanning for all participants was conducted at the University Hospital Basel Switzerland, using a Siemens MAGNETOM Prisma 3 Tesla scanner with a 20-channel radiofrequency head neck coil. Head movement was minimised by foam padding. A whole brain 3-dimensional T1 weighted magnetisation prepared rapid acquisition gradient (MPRAGE) sequence was performed. The acquisition was based on a sagittal matrix of 256 x 256 x 176 and 1mm^3 spatial resolution, with an inversion time of 2000 ms, repetition time of 3.37 ms and flip angle of 8° and bandwidth of 200 Hz/Px. All images were screened by trained neuroradiologists and assessed for radiological abnormalities. Out of the initial sample of 142, 3 participants were not scanned (2 due to previously undisclosed potential metal fragments in eyes) and 1 due to dropout. Structural data of nine participants (5LS/4HS) were excluded due to incidental findings. Results included in this manuscript come from preprocessing performed using *fMRIPrep* 23.0.1 ((39), RRID:SCR_016216), which is based on *Nipype* 1.8.5 ((40, 41); RRID:SCR_002502). A total of 1 T1-weighted (T1w) images were found within the input BIDS dataset.The T1-weighted (T1w) image was corrected for intensity non-uniformity (INU) with N4BiasFieldCorrection(42), distributed with ANTs 2.3.3(43) (RRID:SCR_004757), and used as T1w-reference throughout the workflow. The T1w-reference was then skull-stripped with a *Nipype* implementation of the antsBrainExtraction.sh workflow (from ANTs), using OASIS30ANTs as target template. Brain tissue segmentation of cerebrospinal fluid (CSF), white-matter (WM) and gray-matter (GM) was performed on the brain-extracted T1w using fast (FSL 6.0.5.1:57b01774, RRID:SCR_002823,(44)). Brain surfaces were reconstructed using recon-all (FreeSurfer 7.3.2, RRID:SCR_001847,(45)), and the brain mask estimated previously was refined with a custom variation of the method to reconcile ANTs-derived and FreeSurfer-derived segmentations of the cortical gray-matter of Mindboggle (RRID:SCR_002438,(46)). Volume-based spatial normalisation to one standard space (MNI152NLin2009cAsym) was performed through nonlinear registration with antsRegistration (ANTs 2.3.3), using brain-extracted versions of both T1w reference and the T1w template. The following template was were selected for spatial normalisation and accessed with *TemplateFlow* (23.0.0,(47)): *ICBM 152 Nonlinear Asymmetrical template version 2009c (48)*, RRID:SCR_008796; TemplateFlow ID: MNI152NLin2009cAsym].

FreeSurfer software 7.3+ “recon all” stream and “segment subregions hippo amygdala”, a probabilistic atlas built with ultra-high-resolution ex vivo MRI data (~0.1 mm isotropic) were used to produce an automated segmentation of the hippocampal substructures and the nuclei of the amygdala.

1.5 Arterial Spin Labelling (ASL): preprocessing pipeline

The multi-step approach for pre-processing included: 1) Co-registration of the proton density (Mzero) image to the T1 image**:** This step involved aligning the origins of both images to ensure accurate spatial correspondence. 2) Unified segmentation of the T1 image**:** This process generated a "brain-only" binary mask isolating brain tissue by segmenting the T1 image into different tissue classes. 3) Removal of extracerebral signals from the CBF map**:** The "brain-only" binary mask was applied to the CBF map in T1 space to exclude non-brain signals. 4) Normalisation of the subject's T1 image and the skull-stripped CBF map**:** This was performed using the parameters derived from the unified segmentation process. Finally, the normalised individual CBF maps were spatially smoothed using a 6-mm Gaussian kernel. To normalise for global CBF effects, whole brain group models were mean centred (for more details and a schematic of the preprocessing pipeline see Modinos et al. 2018(49)). A binarised grey matter mask was also applied to all models to restrict effects to grey matter only. To assess potential effects of global CBF (gCBF), on ROI specific group differences, independent samples t-test was performed using the extracted individual gCBF values.

1.6 Proton Magnetic Resonance Spectroscopy (^{1}H-MRS) Methods

Figure S3: ^{1}H-MRS voxel placement

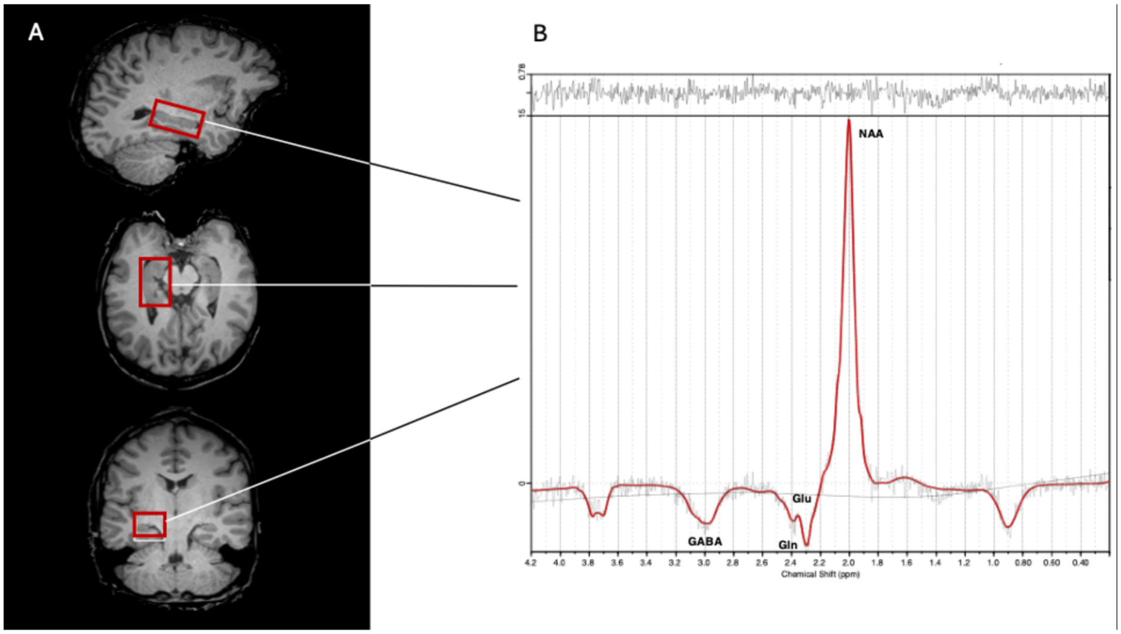

^{1}H-MRS spectra of the left hippocampus were obtained during the same session using water-suppressed MEGA-PRESS (68-millisecond echo time; 1500-milisecond repetition time; 256 averages;

42Hz edit pulse bandwidth, 1.9ppm edit pulse frequency, 4.7ppm edit centre frequency). The hippocampal voxel (25 mm [right-left] × 25 mm [anterior-posterior] × 20 mm [superior- inferior]) was prescribed from the structural T1-weighted scan (supplementary figure 4). MEGA-PRESS difference spectra were analysed using LCModel Version 6.3-1R with a basis set generated using FID-A for the metabolites GABA, glutamate, glutamine, creatine and NAA as well as including a non-simulated, empirically measured macromolecular spectrum (measured from real world data).

1.7 Microbiota data sequencing and processing

The stool samples were collected by the participants at home with the aid of an in-person instruction during the first study session and a remotely accessible tutorial video. The samples were collected at a maximum of seven days before being returned to the study centre and frozen at -80°C. Upon study completion, the samples were shipped from Basel, Switzerland, to Leuven, Belgium, where faecal DNA was extracted according to the protocol outlined by Falony et al. (50). Faecal DNA extraction was performed according to the method described by Falony et al.(50). Briefly, DNA was isolated from 150-200 mg of frozen stool samples using the MagAttract PowerMicrobiome DNA/RNA KF kit (QIAGEN, Hilden, Germany), following the instructions provided by the manufacturer. Amplification of the V4 region of the 16S rRNA genes was conducted using the 515F/806R primer pair, and the PCR products were purified with the QIAquick PCR Purification Kit. Sequencing was executed on an Illumina MiSeq platform (MiSeq Reagent Kit v2, Illumina, San Diego, USA).

1.8 Processing of 16S rRNA Data

Specifically, DNA was extracted from 150–200 mg of the frozen samples, and the V4 region of the 16S ribosomal RNA (rRNA) genes was amplified, purified, and sequenced. Microbial load in the study cohort was assessed using flow cytometry (51). For processing the 16S rRNA data, the amplicon sequences from the 16S rRNA gene were analysed based on the DADA2 pipeline specifications(52). The initial 30 base pairs were removed, and sequence lengths were truncated to 130 bp for forward reads and 200 bp for reverse reads. The sequence error rate correction, dereplication, sample composition inference, and chimaera removal were all conducted using DADA2's standard settings. Taxonomic classification was carried out using the DADA2 RDP implementation ("assignTaxonomy" function within the R package "dada2"), utilising the rdp_train_set_16 as the reference database. The amplicon sequence variant (ASV) annotations were performed with the GTDB_bac120_arc122_ssu_r202_Species training set, applying the "addSpecies" function from the "dada2" R package. Relative abundances were provided at the ASV level and then summarised at the genus level.

1.9 Measurement of Microbial Load

Microbial load in the cohort was assessed by flow cytometry as described previously(53). In short, 200-250 mg aliquots of faecal samples stored at -80°C were diluted in 0.85% NaCl solution (VWR International, Germany) and passed through a sterile syringe filter (5 µm pore size; Sartorius Stedim Biotech GmbH, Göttingen, Germany). Subsequently, 1 mL of the resulting microbial suspension was stained with 1 µL SYBR Green I (diluted 1:100 in DMSO; Thermo Fisher Scientific, Massachusetts, USA) and incubated in the dark at 37°C for 15 minutes. Flow cytometry was conducted using a C6 Accuri flow cytometer (BD Biosciences, New Jersey, USA) following the methodology of Prest et al.10 Fluorescent signals were detected using FL1 (533/30 nm) and FL3 (>670 nm) optical detectors. BD Accuri CFlow software was utilised to distinguish microbial fluorescence events from the background in the faecal samples using FL1/FL3 density plots, with a threshold of 2000 applied to the FL1 channel. Microbial counts were then normalised to the original weight of the aliquots to determine microbial load per gram of faecal matter.

2 Results:

2.1 Cognition

Analysis of the cognitive data showed indications of group differences with small to moderate effect sizes in selective attention and processing speed (VST-R; p^{unc} = .013; Cohen *d* = 0.42), processing speed (TMT-A; p^{unc} = .048; Cohen *d* = 0.34), working memory (TMT-B; p^{unc} = .007; Cohen *d* = 0.46), visuospatial memory (BVMT-R; p^{unc} = .032; Cohen *d* = 0.37) and trends in verbal learning (VLMT I; p^{unc} = .064; Cohen *d* = 0.31) and social cognition (MSCEIT; p^{unc} = .081; Cohen *d* = 0.30), with HS scoring lower than LS, yet did not withstand correction for multiple comparisons (figure 2, and supplementary table 4 for corrected p-values).

Table S3: Clinical Questionnaires

	N (*HS/LS*)	M (*HS/LS*)	SD (*HS/LS*)	t	*df*	*Cohens' d*	*p*	p_{corr}
**3.1 sO-LIFE**								
unusual experiences	69/72	7.45/0.46	1.43/0.50	38.40	83.87	6.52	<.001	<.001
cognitive disorganisation	69/72	8.04/2.65	2.00/2.52	14.10	134.49	2.37	<.001	<.001
introvertive anhedonia	69/72	2.43/1.56	1.90/1.58	2.98	132.47	.50	.003	.113
impulsive nonconformity	69/72	4.39/1.58	1.90/1.46	9.80	127.51	1.65	<.001	<.001
**3.2 SPQ**								
total	68/71	27.28/7.61	12.56/6.83	11.40	102.5	1.95	<.001	<.001
ideas of reference	68/71	4.03/0.82	2.60/1.31	9.13	98.05	1.56	<.001	<.001
excessive social anxiety	68/71	3.75/1.52	2.61/1.66	5.98	113.04	1.02	<.001	<.001
odd beliefs/magical thinking	68/71	1.66/0.20	1.98/0.62	5.82	79.63	1.00	<.001	<.001
unusual perceptual experiences	68/71	3.03/0.32	2.16/0.63	9.92	77.73	1.70	<.001	<.001
odd or eccentric behaviour	68/71	2.90/0.79	2.17/1.40	6.76	113.84	1.15	<.001	<.001
no close friends	68/71	2.31/1.03	1.61/1.61	4.36	132.98	0.74	<.001	<.001
odd speech	68/71	4.66/1.27	2.45/1.63	9.57	115.81	1.63	<.001	<.001
constricted affect	68/71	2.41/1.03	1.97/1.25	4.91	112.73	0.84	<.001	<.001
suspiciousness	68/71	2.53/0.63	1.95/0.97	7.20	97.53	1.23	<.001	<.001
cognitive perceptual	68/71	11.25/1.97	6.69/2.60	10.70	86.04	1.83	<.001	<.001
interpersonal	68/71	11.00/4.21	6.36/4.32	7.33	117.27	1.25	<.001	<.001
disorganised	68/71	7.56/2.06	3.98/2.67	9.54	116.49	1.63	<.001	<.001
**3.3 CAPE**								
total	68/71	93.88/67.30	16.19/10.86	11.41	114.71	1.94	<.001	<.001
positive	68/71	37.63/26.28	8.69/4.23	9.72	96.15	1.66	<.001	<.001
depressed	68/71	22.78/15.72	5.19/3.59	9.30	118.62	1.58	<.001	<.001
negative	68/71	33.47/25.30	7.09/5.32	7.66	124.16	1.30	<.001	<.001
**3.4 DASS**								
total	69/72	15.87/5.21	9.06/4.67	8.71	100.74	1.48	<.001	<.001
depression	69/72	4.46/1.47	3.36/1.67	6.65	98.72	1.13	<.001	<.001
anxiety	69/72	4.20/1.15	3.11/1.41	7.44	93.91	1.26	<.001	<.001
stress	69/72	7.21/2.58	3.93/2.42	8.32	112.26	1.41	<.001	<.001
**3.5 PANAS**								
total	69/72	56.49/51.06	10.59/7.88	3.37	125.49	0.57	<.001	.033
positive affect	69/72	27.84/24.04	6.23/3.73	4.32	110.29	0.73	<.001	.001
negative affect	69/72	28.64/27.01	5.56/4.65	1.81	132.62	0.31	.072	1
**3.6 STAI**								
state	69/72	41.44/35.88	9.28/6.42	7.64	121.46	1.29	<.001	.002
trait	69/72	44.14/31.94	10.94/7.68	4.20	120.44	0.71	<.001	<.001
**3.7 SASPAS**								
total	69/72	12.41/9.11	6.61/5.03	3.42	126.9	0.58	<.001	.028
social anhedonia	69/72	8.21/5.47	4.92/3.71	3.83	126.36	0.65	<.001	.007
physical anhedonia	69/72	4.20/3.64	2.72/1.81	1.48	117.76	0.25	.141	1

Table S4: Cognition

	N (*HS/LS*)	M (*HS/LS*)	SD (*HS/LS*)	t	*df*	*Cohens' d*	*P*	p_{corr}
**4.1 attention/vigilance**								
CPT-IP	68/70	2.50/2.58	0.55/0.61	-0.83	136	0.14	.409	1
**4.2 processing speed**								
VST-R: dots	69/72	12.88/11.99	2.09/2.11	2.52	138.84	0.42	.013	.270
VST-R: words	69/72	13.71/12.76	2.45/2.03	2.52	132.17	0.42	.013	.275
VST-R: colours	69/72	19.83/18.92	4.87/6.74	0.92	129.28	0.16	.357	1
Animal Naming	69/72	26.67/27.93	5.32/4.91	-1.47	139	0.25	.145	1
BACS	69/71	67.93/68.52	8.81/10.17	-0.37	138	0.06	.713	1
SDMT	69/72	66.42/67.28	11.04/10.86	-0.46	138.51	0.08	.643	1
TMT-A	69/72	27.64/25.09	8.24/6.79	1.99	131.85	0.34	.048	1
**4.3 working memory**								
LNS	69/71	16.01/16.39	2.62/2.66	-0.85	138	0.14	.396	1
TMT-B	69/72	56.02/48.67	17.14/14.61	2.73	133.60	0.46	.007	.149
WMS-III: SS	69/71	20.00/20.79	3.16/3.49	-1.40	138	0.24	.164	1
**4.4 verbal learning**								
VLMT 1-5	69/72	63.09/63.88	6.69/6.03	-0.73	136.11	0.12	.464	1
VLMT 6	69/72	13.43/13.74	1.72/1.51	-1.10	135.04	0.19	.272	1
VLMT 7	69/72	13.67/13.75	1.57/1.44	-0.33	136.79	0.06	.743	1
VLMT I	69/72	9.54/10.36	2.60/2.66	-1.86	138.94	0.31	.064	1
**4.5 visuospatial memory**								
BVMT-R	69/72	28.14/29.81	4.97/4.05	-2.17	131.28	0.37	.032	.669
**4.6 reasoning and problem solving**								
NAB MAZES	69/71	22.42/23.18	3.60/3.17	-1.33	134.76	0.22	.186	1
**4.7 social cognition**								
MSCEIT	60/71	109.65/ 112.89	9.59/12.05	-1.76	132.90	0.30	.081	1

Table S5: Hippocampus

	**N** (*HS/LS*)	**M** (*HS/LS*)	**SD** (*HS/LS*)	t	*df*	*Cohens' d*	*p*	p_{corr}
**5.1 structural MRI**								
L Whole Hippocampus	66/70	3166/3188	268/243	-0.52	134	0.09	.605	1
L Whole hippocampal body	66/70	1313/1303	109/104	0.58	134	0.10	.560	1
L Whole hippocampal head	66/70	1853/1886	181/156	-1.16	134	0.20	.250	1
L Hippocampal tail	66/70	613/610	80/73	0.21	134	0.04	.832	1
L subiculum body	66/70	273/272	28/31	0.32	134	0.06	.749	1
L CA1 body	66/70	137/139	25/22	-0.56	128	0.10	.579	1
L subiculum head	66/70	218/219	30/27	-0.24	134	0.04	.809	1
L hippocampal fissure	66/70	151/152	26/25	-0.16	132.65	0.03	.873	1
L presubiculum head	66/70	156/155	20/19	0.39	134	0.07	.694	1
L CA1 head	66/70	564/579	61/53	-1.48	134	0.25	.141	1
L presubiculum body	66/70	190/185	24/25	1.26	134	0.22	.211	1
L parasubiculum	66/70	66/65	12/14	0.46	133.28	0.08	.649	1
L molecular layer HP head	66/70	363/369	37/31	-1.08	134	0.19	.280	1
L molecular layer HP body	66/70	252/250	26/22	0.36	134	0.06	.717	1
L GC ML DG head	66/70	163/167	18/15	-1.46	134	0.25	.148	1
L CA3 body	66/70	94/95	17/15	-0.23	134	0.04	.817	1
L GC ML DG body	66/70	148/146	13/14	0.75	134	0.13	.456	1
L CA4 head	66/70	135/138	15/13	-1.32	134	0.23	.190	1
L CA4 body	66/70	129/129	12/13	0.33	134	0.06	.744	1
L fimbria	66/70	89/87	16/15	1.03	133.37	0.18	.307	1
L CA3 head	66/70	125/129	16/14	-1.5	130.37	0.26	.136	1
L HATA	66/70	63/65	8/8	-1.85	134	0.32	.066	1
R Whole hippocampus	66/70	3170/3180	246/230	-0.25	134	0.04	.801	1
R Whole hippocampal body	66/70	1295/1285	112/93	0.57	134	0.10	.572	1
R Whole hippocampal head	66/70	1876/1896	155/164	-0.74	134	0.13	.462	1
R Hippocampal tail	66/70	640/628	75/75	0.91	134	0.16	.362	1
R subiculum body	66/70	265/258	27/25	1.55	134	0.27	.123	1
R CA1 body	66/70	145/147	23/19	-0.63	134	0.11	.532	1
R subiculum head	66/70	212/210	26/24	0.50	131.53	0.09	.616	1
R hippocampal fissure	66/70	151/150	26/26	0.35	133.30	0.06	.726	1
R presubiculum head	66/70	149/146	16/14	1.21	127.41	0.21	.230	1
R CA1 head	66/70	584/596	56/61	1.18	134	0.20	.241	1
R presubiculum body	66/70	172/166	20/19	1.76	133.05	0.30	.081	1
R parasubiculum	66/70	65/64	13/11	0.55	128.71	0.10	.581	1
R molecular layer HP head	66/70	367/370	32/33	-0.54	134	0.09	.591	1
R molecular layer HP body	66/70	253/251	27/21	0.29	123.28	0.05	.770	1
R GC ML DG head	66/70	166/169	18/17	-1.14	134	0.19	.258	1

R CA3 body	66/70	102/105	19/15	-1.12	134	0.19	.263	1
R GC ML DG body	66/70	146/146	14/13	0.02	134	0.00	.985	1
R CA4 head	66/70	137/139	15/13	-0.90	134	0.15	.368	1
R CA4 body	66/70	129/129	13/12	-0.01	134	0.00	.994	1
R fimbria	66/70	84/82	17/16	0.50	132.79	0.09	.616	1
R CA3 head	66/70	130/135	20/17	-1.53	127.11	0.26	.127	1
R HATA	66/70	65/66	9/10	-0.84	133.1	0.14	.405	1
**5.2 perfusion ASL**								
global mean	66/70	339/328	63/56	1.07	134	0.18	.286	1
global median	66/70	349/336	67/63	1.17	134	0.20	.243	1
global min	66/70	-711/-659	416/464	-0.69	133.66	0.12	.493	1
global max	66/70	1311/1289	399/375	0.33	132.06	0.06	.741	1
Left Hippocampus mean	66/70	414/407	75/72	0.79	134	0.14	.432	1
Right Hippocampus mean	66/70	405/389	71/77	1.28	134	0.22	.202	1
ctx lh parahippocampal mean	66/70	460/452	94/86	0.51	134	0.09	.612	1
ctx rh parahippocampal mean	66/70	446/422	94/88	1.54	134	0.26	.127	1
Left Hippocampus min	66/70	115/95	86/127	1.07	122.26	0.18	.286	1
Right Hippocampus min	66/70	99/58	90/190	1.64	99.99	0.28	.104	1
ctx lh parahippocampal min	66/70	215/200	124/143	0.66	132.97	0.11	.509	1
ctx rh parahippocampal min	66/70	193/137	129/165	2.24	129.86	0.38	.027	.406
Left Hippocampus max	66/70	674/680	164/165	-0.2	133.61	0.03	.845	1
Right Hippocampus max	66/70	662/671	126/191	-0.32	120.52	0.05	.752	1
ctx lh parahippocampal max	66/70	645/640	129/129	0.21	134	0.04	.832	1
**5.3 MR spectroscopy**								
Glu/NAA	67/70	0.88/0.88	0.14/0.14	-0.02	134.93	<.01	.984	1
Glx/NAA	67/71	1.01/0.99	0.27/0.26	0.43	134.60	0.07	.670	1
GABA/NAA	60/62	0.25/0.24	0.06/0.05	1.27	111.32	0.23	.208	1
Glu/GABA	60/62	3.73/3.81	0.99/0.99	-0.49	119.90	0.09	.631	1
Glx/GABA	60/62	4.19/4.27	1.42/1.42	-0.30	119.90	0.05	.767	1

Table S6: Microbiome & Nutrition

	N (*HS/LS*)	M (*HS/LS*)	SD (*HS/LS*)	t	*df*	*Cohens' d*	*p*	p_{corr}
**6.1 alpha diversity**								
inverse simpson	69/71	10.89/11.69	3.94/5.05	-1.05	131.87	0.18	.296	1
observed num. species	69/71	67.88/68.99	16.61/16.76	-0.39	137.94	0.07	.697	1
shannon	69/71	2.93/2.97	0.35/0.36	-0.78	138.00	0.13	.436	1
**6.2 covariates**								
cells/g	69/71	1.45E+11/ 1.43E+11	6.38E+10/ 6.36E+10	-0.64	112.45	0.11	.526	1
BSS interval	68/70	3.16/3.39	1.60/1.52	-0.84	135.08	0.14	.400	1
BSS consistency	68/71	4.18/4.23	1.56/1.47	-0.18	136.31	0.04	.854	1
**6.3 nutrition**								
calcium	56/69	1050/829	882/403	2.31	78.78	0.32	.087	1
carbohydrates	56/69	315/249	210/109	1.73	73.53	0.43	.024	.564
dietary fibres	56/69	33/28	25/18	1.41	96.96	0.26	.161	1
energy (kj)	56/69	2084/1657	1454/665	2.03	73.54	0.38	.046	1
fat	56/69	90/72	67/36	1.80	80.68	0.33	.075	1
iron	56/69	16/12	13/7	2.12	79.41	0.39	.037	.894
magnesium	56/69	438/343	321/170	2	79.64	0.37	.049	1
monosaturated fatty acids	56/69	25/21	19/10	1.65	79.25	0.31	.103	1
phosphorus	56/69	1468/1103	1264/497	2.03	68.78	0.38	.046	1
polyunsaturated fatty acids	56/69	14/11	12/6	1.53	75.01	0.29	.130	1
potassium	56/69	3606/2993	2844/1691	1.42	85.38	0.26	.159	1
protein	56/69	95/69	91/33	2.02	66.91	0.38	.047	1
saturated fatty acids	56/69	39/31	30/19	1.77	88.56	0.33	.085	1
sodium	56/69	438/343	1695/897	1.88	79.53	0.26	.064	1
sugar	56/69	103/84	91/56	1.4	88.98	0.25	.165	1
vitamin B12 cobalamin	56/69	6/4	9/3	1.34	67.95	0.25	.186	1
vitamin C ascorbic acid	56/69	164/146	166/143	0.61	109.06	0.11	.545	1
vitamin D calciferol	56/69	5/4	7/3	1.21	73.80	0.23	.230	1

Figure S4: Significant clinical correlations biological markers within HS

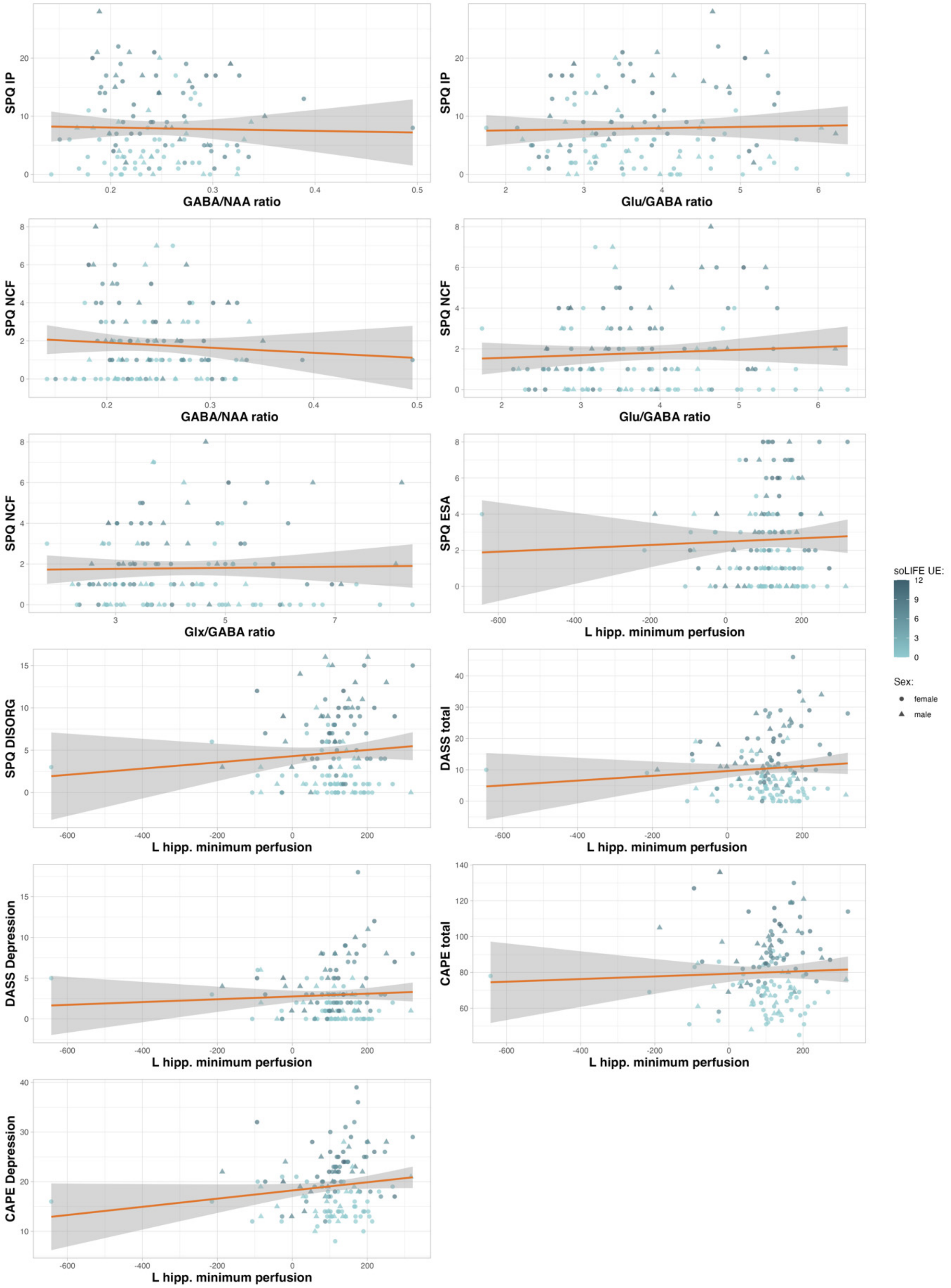

Figure S5: Significant correlations between hippocampus modalities across the entire sample (HS&LS)

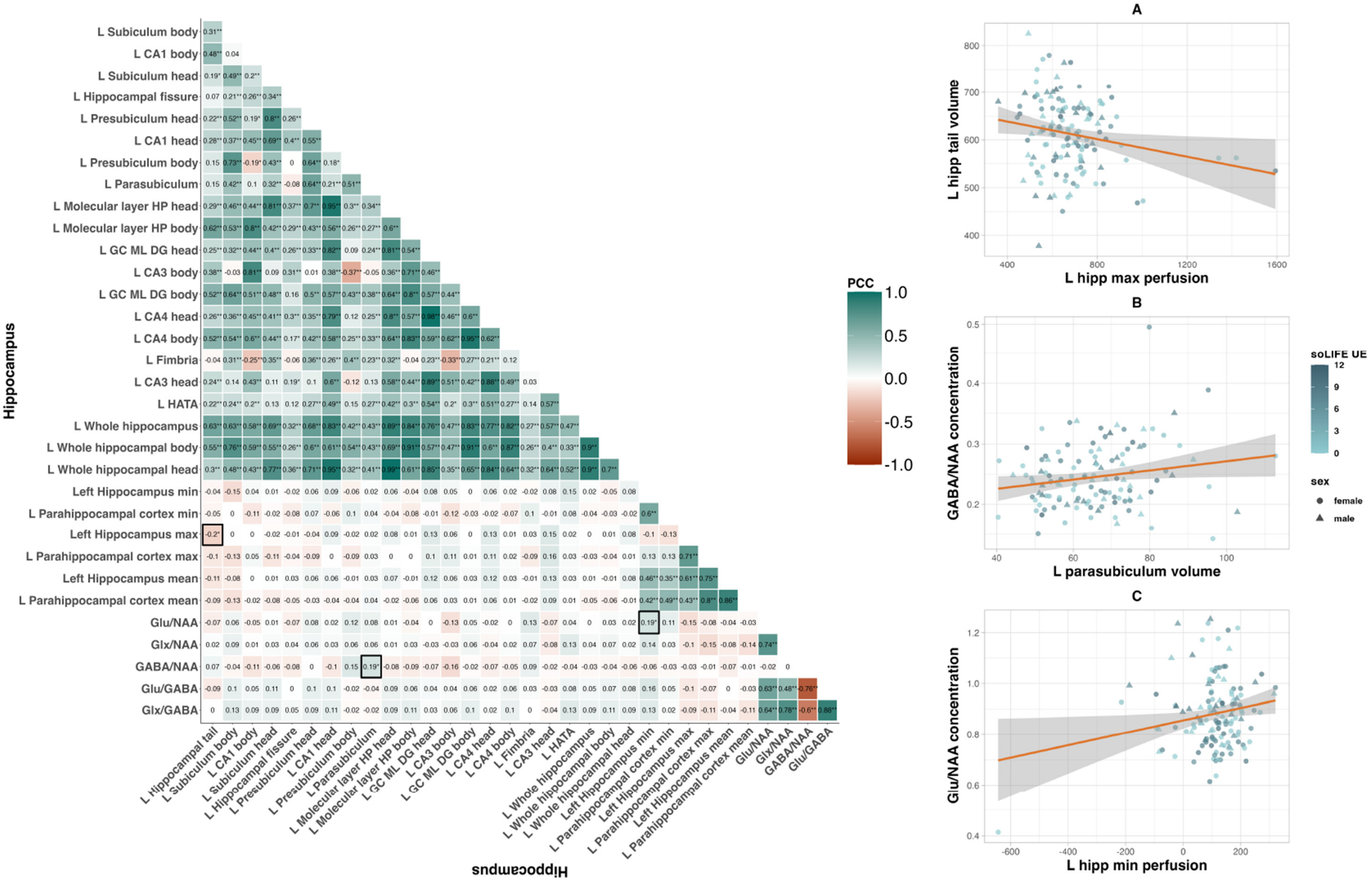

2.2 Gut microbiota ecosystem-level analysis

Using Bray-Curtis dissimilarity, we observed that the HS group did not contribute to microbiota variation at the genus level (dbRDA adjusted $R^2 < 0.001$, PERMANOVA $p = 0.947$) (supplementary figure 6A), nor at the ASV level (dbRDA adjusted $R^2 = 0.001$, PERMANOVA $p = 0.253$) (supplementary figure 6B). The BSS (proxy of transit time) had a significant contribution to gut microbiota compositional variation (Genus: dbRDA adjusted $R^2 = 0.017$, PERMANOVA, $p = 0.001$; ASVs: dbRDA adjusted $R^2 = 0.012$; PERMANOVA, $p = 0.001$) (supplementary figure 6C). The distribution of enterotypes did not differ between schizotypy groups ($\chi 2 = 3.87$, df = 3, $p = .283$) (supplementary figure 7). The comparison of alpha diversity measures of species abundance (inverse simpson; $p^{unc} = .296$; Cohen $d = 0.18$), species distribution (shannon; $p^{unc} = .436$ Cohen $d = 0.13$), number of observed species ($p^{unc} = .697$; Cohen $d = 0.07$) (supplementary figure 8, for corrected p-values see supplementary table 6), and total cell count per gram (cells/g; $p^{unc} = .862$; Cohen $d = 0.03$) did not reveal statistically significant group differences (supplementary table 6).

Figure S6. Comparison of β diversity analysis between high positive schizotypy (HS) and low positive schizotypy (LS) cohort. Microbiota variation at A) genus level, and B) ASVs level. C) Contribution of BSS to gut microbiota compositional variation at BSS and ASVs level.

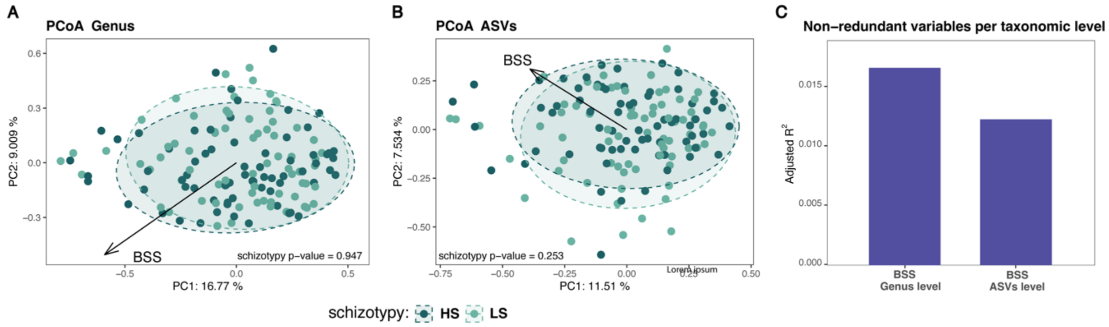

Table S7: Microbiome Taxa

	N (*HS/LS*)	median (*HS*)	median (*LS*)	IQR (*HS*)	IQR (*LS*)	*Z*	*p*	p_{corr}
**Genus**								
992a	69/71	5.75E+09	5.75E+09	7.81E+09	6.78E+09	0.04	.970	1
Acidaminococcus	69/71	0	76182929	1.9E+08	4.38E+08	-1.74	.062	1
Agathobacter	69/71	4.53E+09	3.43E+09	8.87E+09	1.05E+10	0.36	.720	1
Agathobaculum	69/71	5.71E+08	5.71E+08	7.24E+08	7.43E+08	0.29	.774	1
Alistipes	69/71	2.82E+09	3.31E+09	5.18E+09	4.65E+09	-0.63	.529	1
Alistipes_A	69/71	0	76181982	1.14E+08	3.62E+08	-1.89	.046	1
Anaeromassilibacillus	69/71	0	0	0	38087563	-0.43	.572	1
Anaerorhabdus	69/71	0	0	0	38091877	-1.62	.046	1
Anaerosporobacter	69/71	0	0	0	0	0.05	.947	1
Anaerotignum	69/71	1.14E+08	1.52E+08	1.52E+08	2.86E+08	-1.69	.090	1
Anaerotruncus	69/71	0	0	0	0	-0.53	.461	1
Bacteroides	69/71	3.01E+09	3.54E+09	4.88E+09	4.38E+09	-0.53	.599	1
Bacteroides_B	69/71	8.38E+08	4.57E+08	3.01E+09	2.48E+09	1.53	.125	1
Barnesiella	69/71	1.52E+08	1.52E+08	2.67E+08	3.24E+08	-0.09	.927	1
Bifidobacterium	69/71	2.32E+09	2.21E+09	4.65E+09	3.98E+09	0.60	.548	1
Bittarella	69/71	1.33E+09	2.55E+09	3.7E+09	5.83E+09	-1.48	.138	1
Blautia	69/71	0	0	0	76180045	-1.23	.111	1
Blautia_A	69/71	5.87E+09	6.17E+09	5.71E+09	6.15E+09	-1.03	.303	1
Butyricimonas	69/71	0	38087406	76179261	1.14E+08	-1.09	.235	1
CAG-110	69/71	1.52E+08	2.67E+08	6.1E+08	7.62E+08	-0.57	.567	1
CAG-24	69/71	1.52E+08	1.14E+08	3.05E+08	2.48E+08	1	.310	1
CAG-354	69/71	76168315	38089072	3.05E+08	7.05E+08	-0.24	.800	1
CAG-41	69/71	4.19E+08	3.43E+08	4.95E+08	5.9E+08	0.82	.414	1
CAG-45	69/71	0	0	0	1.14E+08	-0.49	.533	1
CAG-495	69/71	0	0	76193853	1.14E+08	0.10	.911	1
CAG-56	69/71	4.95E+08	4.19E+08	1.41E+09	1.68E+09	-0.01	.992	1
CAG-65	69/71	2.29E+08	1.9E+08	7.62E+08	7.62E+08	0.92	.357	1
CAG-81	69/71	3.43E+08	2.29E+08	4.19E+08	4.19E+08	1.06	.287	1
CHKCI001	69/71	1.9E+08	1.9E+08	3.05E+08	4E+08	0.70	.484	1
Clostridium	69/71	0	0	38092647	76179122	-0.91	.308	1
Clostridium_A	69/71	0	0	76178758	1.14E+08	-0.63	.479	1
Clostridium_AJ	69/71	76184507	76178607	2.29E+08	1.52E+08	0.37	.709	1
Clostridium_N	69/71	0	0	1.14E+08	95225795	-0.24	.783	1
Collinsella	69/71	5.33E+08	4.57E+08	1.37E+09	1.31E+09	0.60	.547	1
Coprobacter	69/71	38094695	76174812	1.9E+08	3.62E+08	-0.36	.714	1

Coprococcus	69/71	76180472	1.9E+08	2.17E+09	1.62E+09	0.17	.859	1
Desulfovibrio	69/71	0	0	0	38092951	-0.20	.796	1
Dialister	69/71	6.09E+08	4.57E+08	1.52E+09	1.5E+09	0.40	.688	1
Dorea	69/71	1.9E+09	2.32E+09	2.44E+09	2.32E+09	-1.72	.086	1
DTU053	69/71	4.57E+08	4.95E+08	1.03E+09	1.24E+09	-0.04	.969	1
Dubosiella	69/71	0	0	76180472	0	0.48	.519	1
Eggerthella	69/71	0	0	38084776	0	0.17	.822	1
Emergencia	69/71	0	0	76173921	76174353	-0.05	.960	1
Enterorhabdus	69/71	1.52E+08	1.52E+08	4.19E+08	3.05E+08	0.13	.900	1
Erysipelatoclostridium	69/71	8.76E+08	9.14E+08	1.87E+09	2.21E+09	-0.40	.694	1
Escherichia	69/71	0	0	76176419	38094236	-0.08	.932	1
Eubacterium_D	69/71	0	76173734	1.52E+08	1.71E+08	-2.09	.031	1
Eubacterium_E	69/71	1.26E+09	1.3E+09	1.52E+09	1.56E+09	-0.77	.442	1
Eubacterium_F	69/71	76169112	38093802	4.95E+08	3.62E+08	0.16	.868	1
Eubacterium_R	69/71	4.19E+08	3.81E+08	8.38E+08	9.9E+08	-0.20	.843	1
Faecalibacterium	69/71	2.1E+10	1.79E+10	2.4E+10	2.6E+10	1.05	.294	1
Flavonifractor	69/71	1.14E+08	1.52E+08	3.05E+08	4.38E+08	-1.33	.182	1
Fusicatenibacter	69/71	1.41E+09	1.45E+09	1.6E+09	1.94E+09	0.06	.950	1
GCA-900066135	69/71	1.14E+08	1.14E+08	76213147	2.09E+08	-0.61	.539	1
GCA-900066575	69/71	38092240	38090991	1.52E+08	76185759	0.49	.616	1
Gordonibacter	69/71	0	0	0	1.14E+08	-2.23	.006	.527
Haemophilus_D	69/71	0	0	38089060	0	0.64	.399	1
Intestinibacter	69/71	38092880	38091653	1.14E+08	76186705	0.75	.444	1
Intestinimonas	69/71	1.9E+08	2.67E+08	4.19E+08	2.67E+08	-1.21	.226	1
Lachnospira	69/71	1.07E+09	9.9E+08	1.37E+09	1.58E+09	0.62	.537	1
Massilioclostridium	69/71	0	0	76174797	76180108	0.16	.849	1
Massiliomicrobiota	69/71	0	0	0	38092184	-0.98	.218	1
Odoribacter	69/71	38097124	76185147	1.52E+08	1.71E+08	-1.89	.056	1
Oscillibacter	69/71	4.95E+08	7.24E+08	1.18E+09	1.26E+09	-0.98	.328	1
Parabacteroides	69/71	2.67E+08	1.9E+08	3.81E+08	3.81E+08	1.42	.156	1
Paraprevotella	69/71	0	0	1.14E+08	1.71E+08	0.07	.933	1
Parasutterella	69/71	0	0	76185295	76181530	0.06	.948	1
PeH17	69/71	0	0	0	57135512	-0.89	.268	1
Prevotella	69/71	0	0	4.95E+08	1.71E+09	-0.60	.485	1
Romboutsia	69/71	38087224	38087509	76178758	76188845	-0.50	.598	1
Roseburia	69/71	3.85E+09	3.2E+09	8.95E+09	6.51E+09	0.70	.486	1
Ruminiclostridium_E	69/71	38082183	38096274	4.57E+08	5.9E+08	-0.41	.663	1
Ruminococcus_B	69/71	4.15E+09	4.57E+09	5.6E+09	5.31E+09	-1.04	.297	1

Ruminococcus_C	69/71	1.14E+08	1.52E+08	6.48E+08	8.19E+08	-0.39	.686	1
Ruminococcus_D	69/71	2.67E+08	1.9E+08	9.9E+08	1.01E+09	0.35	.721	1
Ruminococcus_E	69/71	1.52E+09	1.68E+09	4.23E+09	4.69E+09	0.02	.985	1
Ruthenibacterium	69/71	0	0	76180444	76188193	-0.17	.846	1
Senegalimassilia	69/71	0	0	76178758	38086667	0.55	.497	1
Soleaferrea	69/71	0	0	0	38092102	-1.42	.060	1
Streptococcus	69/71	1.14E+08	76192050	2.29E+08	2.67E+08	-0.65	.514	1
Subdoligranulum	69/71	1.3E+09	1.29E+09	2.9E+09	2.36E+09	0.93	.353	1
Sutterella	69/71	5.71E+08	4.95E+08	1.18E+09	9.52E+08	0.23	.820	1
Terrisporobacter	69/71	0	0	38089140	0	0.85	.289	1
TF01-11	69/71	76086957	38096270	3.81E+08	4.76E+08	-0.26	.782	1
Tidjanibacter	69/71	0	0	0	0	-0.46	.521	1
Turicibacter	69/71	0	0	38091223	38095940	-0.21	.805	1
UBA1191	69/71	1.14E+08	76185147	2.29E+08	2.09E+08	0.14	.890	1
UBA5808	69/71	0	0	38098086	1.52E+08	-1.01	.247	1

Figure S7: Gut microbiota enterotype distribution

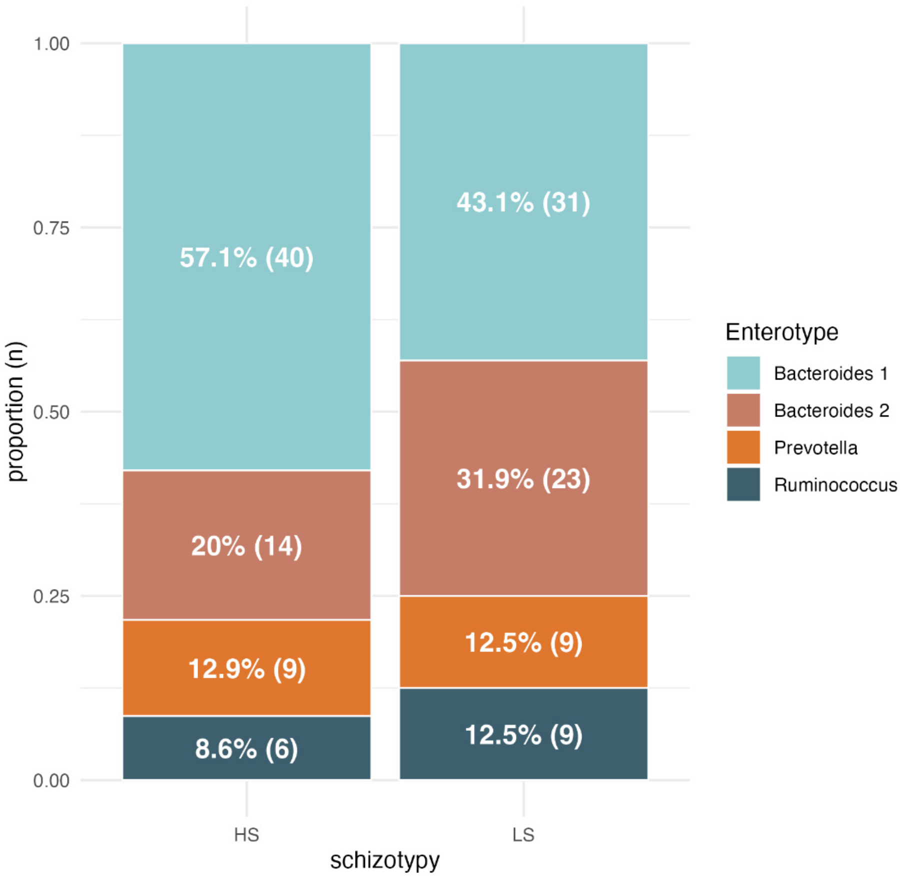

Figure S8: Group differences genus level taxa

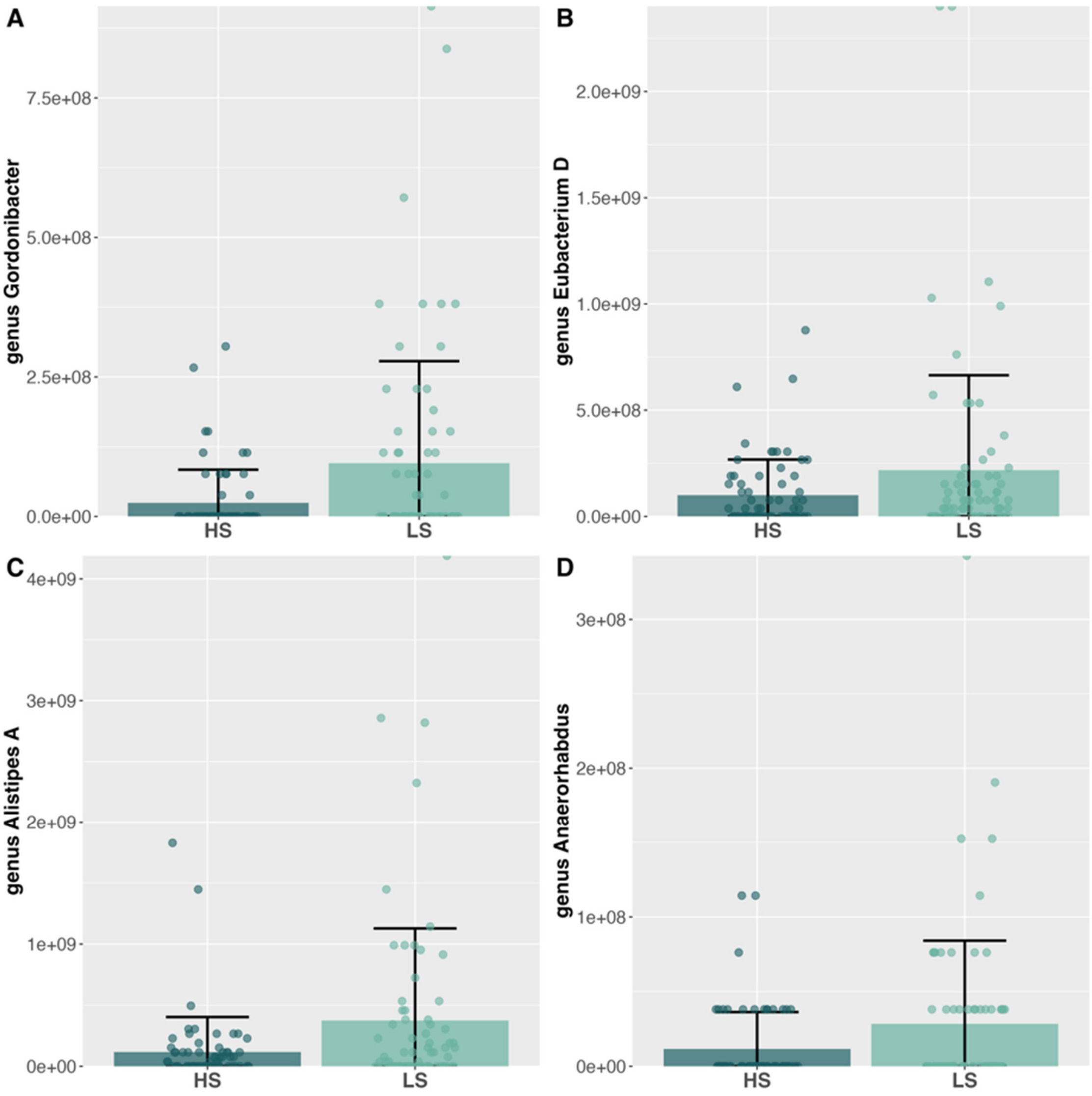

Note. Significant group differences of four genus level A) Gordonibacter, B) Eubacterium D, C) Alistipes A, and D) Anaerorhabdus taxa (before correction). Error bars represent ±1 standard deviation from the group mean. Individual data points are displayed as jittered dots to illustrate the distribution within each group.

Figure S9: Relative versus quantitative microbme profiling in high schizotypy (HS) and low schizotypy (LS).

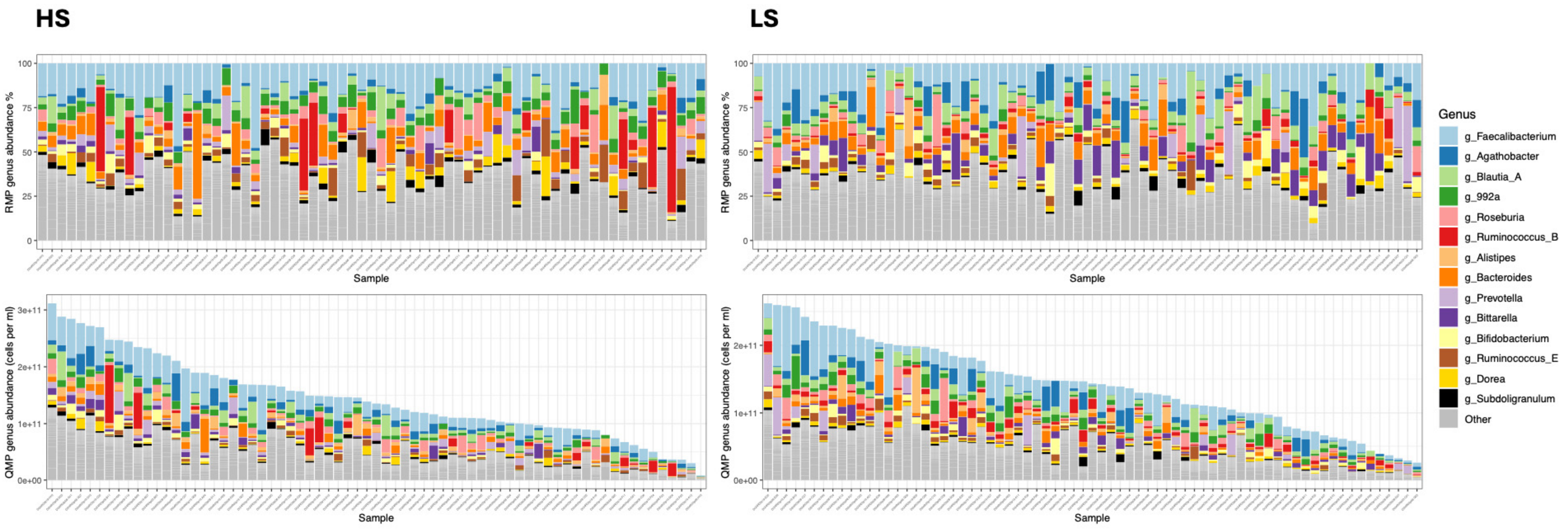

**Microbial Taxa QMP** Genus:	**N**	**median** *(HS)*	**median** *(LS)*		**IQR** *(HS)*	**IQR** *(LS)*	*Z*	*p*	p_{corr}
Faecalibacterium	69/71	2.1E+10	1.79E+10		2.4E+10	2.6E+10	1.05	.294	1
Agathobacter	69/71	4.53E+09	3.43E+09		8.87E+09	1.05E+10	0.36	.720	1
Blautia A	69/71	5.87E+09	6.17E+09		5.71E+09	6.15E+09	-1.03	.303	1
992a	69/71	5.75E+09	5.75E+09		7.81E+09	6.78E+09	0.04	.970	1
Roseburia	69/71	3.85E+09	3.2E+09		8.95E+09	6.51E+09	0.70	.486	1
Ruminococcus B	69/71	4.15E+09	4.57E+09		5.6E+09	5.31E+09	-1.04	.297	1
Alistipes	69/71	2.82E+09	3.31E+09		5.18E+09	4.65E+09	-0.63	.529	1
Bacteroides	69/71	3.01E+09	3.54E+09		4.88E+09	4.38E+09	-0.53	.599	1
Prevotella	69/71	0	0		4.95E+08	1.71E+09	-0.60	.485	1
Bittarella	69/71	1.33E+09	2.55E+09		3.7E+09	5.83E+09	-1.48	.138	1
Bifidobacterium	69/71	2.32E+09	2.21E+09		4.65E+09	3.98E+09	0.60	.548	1
Ruminococcus E	69/71	38082183	38096274		4.57E+08	5.9E+08	0.53	.663	1
Dorea	69/71	1.9E+09	2.32E+09		2.44E+09	2.32E+09	-1.72	.086	1
Subdoligranulum	69/71	1.3E+09	1.29E+09		2.9E+09	2.36E+09	0.93	.353	1

Note: Genus-level faecal microbiome composition of HS (N = 69) and LS (N = 71) study cohort participants. Samples are ordered according to microbial load highest to lowest. The 15 most abundant genera are depicted, with all others pooled into "Other.

Table S8: Enterotypes pw comparisons

	U	Z	*r*	*p*	p_{corr}
**L presubiculum head**					
Bact 1 vs. Bact 2	1647	2.76	0.66	.006	.011
Bact 1 vs. Prevotella	903	3.22	0.75	.001	.007
Bact 1 vs. Ruminococcus	421	-0.59	0.45	.553	.553
Bact 2 vs. Prevotella	367	0.6	0.55	.548	.553
Bact 2 vs. Ruminococcus	142	-2.46	0.27	.014	.021
Prevotella vs. Ruminococcus	45	-3.06	0.18	.002	.007
**L presubiculum body**					
Bact 1 vs. Bact 2	1700	3.12	0.69	.002	.011
Bact 1 vs. Prevotella	857	2.73	0.71	.006	.019
Bact 1 vs. Ruminococcus	481	-0.14	0.51	.886	.886
Bact 2 vs. Prevotella	364	0.55	0.55	.584	.701
Bact 2 vs. Ruminococcus	165	-1.97	0.32	.048	.097
Prevotella vs. Ruminococcus	77	-1.84	0.31	.065	.098
**Glx/GABA**					
Bact 1 vs. Bact 2	947	-0.67	0.46	.503	.503
Bact 1 vs. Prevotella	328	-1.86	0.35	.063	.095
Bact 1 vs. Ruminococcus	525	2.62	0.74	.009	.019
Bact 2 vs. Prevotella	228	-1.05	0.41	.296	.355
Bact 2 vs. Ruminococcus	317	2.6	0.75	.009	.019
Prevotella vs. Ruminococcus	161	2.99	0.84	.003	.017
**Glu/GABA**					
Bact 1 vs. Bact 2	927	-0.82	0.45	.412	.412
Bact 1 vs. Prevotella	320	-1.96	0.34	.050	.075
Bact 1 vs. Ruminococcus	509	2.37	0.72	.018	.036
Bact 2 vs. Prevotella	223	-1.15	0.4	.251	.302
Bact 2 vs. Ruminococcus	319	2.65	0.76	.008	.024
Prevotella vs. Ruminococcus	158	2.86	0.82	.004	.024
**Glx/NAA**					
Bact 1 vs. Bact 2	1387	0.73	0.54	.468	.468
Bact 1 vs. Prevotella	506	-1.2	0.41	.230	.276
Bact 1 vs. Ruminococcus	686	2.46	0.71	.014	.039

Bact 2 vs. Prevotella	235	-1.75	0.35	.080	.120
Bact 2 vs. Ruminococcus	370	2.33	0.71	.019	.039
Prevotella vs. Ruminococcus	203	2.91	0.81	.004	.021
**GABA/NAA**					
Bact 1 vs. Bact 2	1189	1.22	0.58	.224	.269
Bact 1 vs. Prevotella	585	1.45	0.62	.146	.219
Bact 1 vs. Ruminococcus	194	-2.45	0.27	.014	.029
Bact 2 vs. Prevotella	302	0.44	0.54	.663	.663
Bact 2 vs. Ruminococcus	103	-2.6	0.25	.009	.028
Prevotella vs. Ruminococcus	39	-2.62	0.2	.009	.028

References

1. Taylor EM. Cambridge, MA and London, England: Harvard University Press; 1959.
2. Helmstaedter C, & Durwen, H. F. . VLMT: Verbaler Lern- und Merkfähigkeitstest: Ein praktikables und differenziertes Instrumentarium zur Prüfung der verbalen Gedächtnisleistungen. Schweizer Archiv für Neurologie, Neurochirurgie und Psychiatrie. 1990;141(1):21-30.
3. Lehrl S. Mehrfachwahl-Wortschatz-Intelligenztest : MWT-B. 5., unveränd. Aufl. ed. Balingen: Spitta; 2005.
4. Stroop JR. Studies of interference in serial verbal reactions. Journal of Experimental Psychology. 1935;18(6):643-62.
5. Regard M. Cognitive Rigidity and Flexibility [microform] : a Neuropsychological Study: Thesis (Ph.D.)--University of Victoria; 1981.
6. Strauss JL, Hayes AM, Johnson SL, Newman CF, Brown GK, Barber JP, et al. Early alliance, alliance ruptures, and symptom change in a nonrandomized trial of cognitive therapy for avoidant and obsessive-compulsive personality disorders. J Consult Clin Psychol. 2006;74(2):337-45.
7. Smith A. Symbol Digit Modalities Test (SDMT). Manual (Revised) Los Angeles: Western Psychological Services. 1982.
8. Hanssen M, Peeters F, Krabbendam L, Radstake S, Verdoux H, van Os J. How psychotic are individuals with non-psychotic disorders? Social Psychiatry and Psychiatric Epidemiology. 2003;38(3):149-54.
9. Stefanis NC, Hanssen M, Smirnis NK, Avramopoulos DA, Evdokimidis IK, Stefanis CN, et al. Evidence that three dimensions of psychosis have a distribution in the general population. Psychological Medicine. 2002;32(2):347-58.
10. Mossaheb N, Becker J, Schaefer MR, Klier CM, Schloegelhofer M, Papageorgiou K, et al. The Community Assessment of Psychic Experience (CAPE) questionnaire as a screening-instrument in the detection of individuals at ultra-high risk for psychosis. Schizophr Res. 2012;141(2-3):210-4.
11. Konings M, Bak M, Hanssen M, van Os J, Krabbendam L. Validity and reliability of the CAPE: a self-report instrument for the measurement of psychotic experiences in the general population. Acta Psychiatr Scand. 2006;114(1):55-61.
12. Lovibond PF, Lovibond SH. The structure of negative emotional states: comparison of the Depression Anxiety Stress Scales (DASS) with the Beck Depression and Anxiety Inventories. Behav Res Ther. 1995;33(3):335-43.
13. Watson DB, Clark LA, Tellegen A. Development and validation of brief measures of positive and negative affect: the PANAS scales. Journal of personality and social psychology. 1988;54 6:1063-70.
14. Krohne HW, Egloff B, Kohlmann C-W, Tausch A. 2016.
15. Chapman LJ, Chapman JP, Raulin ML. Scales for physical and social anhedonia. J Abnorm Psychol. 1976;85(4):374-82.
16. Burgdörfer G, Hautzinger M. [Psychological and social anhedonia. Evaluation of a research instrument for measuring a basic psychopathologic disorder]. Eur Arch Psychiatry Neurol Sci. 1987;236(4):223-9.
17. Mason O, Linney Y, Claridge G. Short scales for measuring schizotypy. Schizophrenia Research. 2005;78(2):293-6.
18. Linney YM, Murray RM, Peters ER, MacDonald AM, Rijsdijk F, Sham PC. A quantitative genetic analysis of schizotypal personality traits. Psychological Medicine. 2003;33(5):803-16.
19. Mason O, Linney Y, Claridge G. Short scales for measuring schizotypy. Schizophr Res. 2005;78(2-3):293-6.
20. Klein C, Andresen B, Jahn T. Erfassung der schizotypen Persönlichkeit nach DSM-III-R: Psychometrische Eigenschaften einer autorisierten deutschsprachigen Übersetzung des "Schizotypal Personality Questionnaire" (SPQ) von Raine. / Psychometric assessment of the schizotypal

personality according to DSM-III-R criteria: Psychometric properties of an authorized German translation of Raine's "Schizotypal Personality Questionnaire" (SPQ). Diagnostica. 1997;43:347-69.
21. Raine A. The SPQ: a scale for the assessment of schizotypal personality based on DSM-III-R criteria. Schizophr Bull. 1991;17(4):555-64.
22. Spielberger CD. State-trait anxiety inventory for adults. Sampler set: Palo Alto, CA : Mind Garden, [1983] ©1983; 1983.
23. Steinemann N, Grize L, Ziesemer K, Kauf P, Probst-Hensch N, Brombach C. Relative validation of a food frequency questionnaire to estimate food intake in an adult population. Food & Nutrition Research. 2017;61(0).
24. Holzmann M SN, Brombach C. Comparison of the short form of a Swiss Food Frequency Questionnaire with its long form and analysis of the application of an online, image-based nutrition record. Ernahrungs Umschau. 2024;71(10):120-4.
25. Lewis SJ, Heaton KW. Stool form scale as a useful guide to intestinal transit time. Scand J Gastroenterol. 1997;32(9):920-4.
26. Lewis SJ, Heaton KW. Stool Form Scale as a Useful Guide to Intestinal Transit Time. Scandinavian Journal of Gastroenterology. 2009;32(9):920-4.
27. Spreen O, Strauss E. A Compendium of Neuropsychological Tests: Administration, Norms, and Commentary: Oxford University Press, USA; 1998.
28. <nuechterlein-et-al-2008-the-matrics-consensus-cognitive-battery-part-1-test-selection-reliability-and-validity.pdf>.
29. Benedict RHB. Brief Visuospatial Memory Test--revised: PAR; 1997.
30. Keefe RSE, Goldberg TE, Harvey PD, Gold JM, Poe MP, Coughenour L. The Brief Assessment of Cognition in Schizophrenia: reliability, sensitivity, and comparison with a standard neurocognitive battery. Schizophrenia Research. 2004;68(2):283-97.
31. Cornblatt BA, Risch NJ, Faris G, Friedman D, Erlenmeyer-Kimling L. The continuous performance test, identical pairs version (CPT-IP): I. new findings about sustained attention in normal families. Psychiatry Research. 1988;26(2):223-38.
32. Gold JM, Carpenter C, Randolph C, Goldberg TE, Weinberger DR. Auditory Working Memory and Wisconsin Card Sorting Test Performance in Schizophrenia. Archives of General Psychiatry. 1997;54(2):159-65.
33. Mayer JD, Saloyev, P., & Caruso, D. Mayer-Salovey-Caruso Emotional Intelligence Test (MSCEIT). MHS Publishers. 2002.
34. White T, & Stern, R. A. Neuropsychological Assessment Battery: Psychometric and Technical Manual. Psychological Assessment Resources. 2003.
35. Troyer AK, Leach, L., & Strauss, E. Aging and Response Inhibition: Normative Data for the Victoria Stroop Test. Aging, Neuropsychology, and Cognition. 2006;13(1):20-35.
36. Tombaugh TN. Trail Making Test A and B: Normative data stratified by age and education. Archives of Clinical Neuropsychology. 2004;19(2):203-14.
37. Llinàs-Reglà J, Vilalta-Franch J, López-Pousa S, Calvó-Perxas L, Torrents Rodas D, Garre-Olmo J. The Trail Making Test. Assessment. 2017;24(2):183-96.
38. Wechsler D. WMS-III: Wechsler Memory Scale Administration and Scoring Manual: Psychological Corporation; 1997.
39. Esteban O, Markiewicz CJ, Blair RW, Moodie CA, Isik AI, Erramuzpe A, et al. fMRIPrep: a robust preprocessing pipeline for functional MRI. Nature Methods. 2019;16(1):111-6.
40. Gorgolewski K, Burns CD, Madison C, Clark D, Halchenko YO, Waskom ML, et al. Nipype: A Flexible, Lightweight and Extensible Neuroimaging Data Processing Framework in Python. Frontiers in Neuroinformatics. 2011;5.
41. Gorgolewski KJ, Oscar Esteban, Christopher J. Markiewicz, Erik Ziegler, David Gage Ellis, Michael Philipp Notter, Dorota Jarecka, et al. . “Nipype.”. 2018.
42. Tustison NJ, Avants BB, Cook PA, Zheng Y, Egan A, Yushkevich PA, et al. N4ITK: improved N3 bias correction. IEEE Trans Med Imaging. 2010;29(6):1310-20.

43. Avants BB, Epstein CL, Grossman M, Gee JC. Symmetric diffeomorphic image registration with cross-correlation: Evaluating automated labeling of elderly and neurodegenerative brain. Medical Image Analysis. 2008;12(1):26-41.
44. Zhang Y, Brady M, Smith S. Segmentation of brain MR images through a hidden Markov random field model and the expectation-maximization algorithm. IEEE Transactions on Medical Imaging. 2001;20(1):45-57.
45. Dale AM, Fischl B, Sereno MI. Cortical Surface-Based Analysis: I. Segmentation and Surface Reconstruction. Neuroimage. 1999;9(2):179-94.
46. Klein A, Ghosh SS, Bao FS, Giard J, Häme Y, Stavsky E, et al. Mindboggling morphometry of human brains. PLOS Computational Biology. 2017;13(2):e1005350.
47. Ciric R, Thompson WH, Lorenz R, Goncalves M, MacNicol EE, Markiewicz CJ, et al. TemplateFlow: FAIR-sharing of multi-scale, multi-species brain models. Nature Methods. 2022;19(12):1568-71.
48. Fonov VS, Evans AC, McKinstry RC, Almli CR, Collins DL. Unbiased nonlinear average age-appropriate brain templates from birth to adulthood. Neuroimage. 2009;47:S102.
49. Modinos G, Egerton A, McMullen K, McLaughlin A, Kumari V, Barker GJ, et al. Increased resting perfusion of the hippocampus in high positive schizotypy: A pseudocontinuous arterial spin labeling study. Hum Brain Mapp. 2018;39(10):4055-64.
50. Falony G, Joossens M, Vieira-Silva S, Wang J, Darzi Y, Faust K, et al. Population-level analysis of gut microbiome variation. Science. 2016;352(6285):560-4.
51. Vandeputte D, Kathagen G, D'Hoe K, Vieira-Silva S, Valles-Colomer M, Sabino J, et al. Quantitative microbiome profiling links gut community variation to microbial load. Nature. 2017;551(7681):507-11.
52. Callahan BJ, McMurdie PJ, Rosen MJ, Han AW, Johnson AJA, Holmes SP. DADA2: High-resolution sample inference from Illumina amplicon data. Nature Methods. 2016;13(7):581-3.
53. Vandeputte D, Kathagen G, D’hoe K, Vieira-Silva S, Valles-Colomer M, Sabino J, et al. Quantitative microbiome profiling links gut community variation to microbial load. Nature. 2017;551(7681):507-11.
